# Diet-Wide Association, Genetic Susceptibility and Colorectal Cancer Risk: A Prospective Cohort Study

**DOI:** 10.3390/nu15224801

**Published:** 2023-11-16

**Authors:** Dongqing Jin, Ying Lu, Wei Wu, Fangyuan Jiang, Zihan Li, Liying Xu, Rongqi Zhang, Xue Li, Dong Chen

**Affiliations:** 1 Department of Colorectal Surgery, The First Affiliated Hospital, Zhejiang University School of Medicine, Hangzhou 310000, China; 13396567129@163.com; 2 Department of Big Data in Health Science School of Public Health, and Centre of Clinical Big Data and Analytics of the Second Affiliated Hospital, Zhejiang University School of Medicine, Hangzhou 310000, China; luying0303@126.com (Y.L.);; 3 Department of Medical Oncology, The First Affiliated Hospital, Zhejiang University School of Medicine, Hangzhou 310000, China; 4 Centre for Global Health, Usher Institute, University of Edinburgh, Edinburgh EH8 9YL, UK; 5 The Key Laboratory of Intelligent Preventive Medicine of Zhejiang Province, Hangzhou 310058, China

**Keywords:** dietary, calcium, colorectal, CRC, UK Biobank

## Abstract

Background: Both genetic and dietary factors play significant roles in the etiology of colorectal cancer (CRC). To evaluate the relationship between certain food exposures and the risk of CRC, we carried out a large-scale association analysis in the UK Biobank. Methods: The associations of 139 foods and nutrients’ intake with CRC risk were assessed among 118,210 participants. A polygenic risk score (PRS) of CRC was created to explore any interaction between dietary factors and genetic susceptibility in CRC risk. The hazard ratio (HR) and 95% confidence interval (CI) of CRC risk linked to dietary variables and PRS were estimated using Cox regression models. Multiple comparisons were corrected using the error discovery rate (FDR). Results: During a mean follow-up of 12.8 years, 1466 incidents of CRC were identified. In the UK Biobank, alcohol and white bread were associated with increased CRC risk, and their HRs were 1.08 (95% CI: 1.03–1.14; FDRP = 0.028) and 1.10 (95% CI: 1.05–1.16; FDRP = 0.003), whereas dietary fiber, calcium, magnesium, phosphorus, and manganese intakes were inversely associated. We found no evidence of any PRS–nutrient interaction relationship in relation to CRC risk. Conclusions: Our results show that higher intakes of alcohol and white bread are associated with increased CRC risk, whilst dietary fiber, calcium, magnesium, phosphorus, and manganese are inversely associated.

## 1. Introduction

The most common cause of cancer-related fatalities worldwide is still colorectal cancer (CRC) [1]. Approximately 60–65% of CRC cases are episodic, with a large proportion of potentially modifiable risk factors, such as epigenetic aberrations [2]. A sedentary lifestyle, obesity, smoking, excessive alcohol use, a high intake of processed meats, and a poor intake of fruits and vegetables are some of the lifestyle factors that contribute to CRC [3]. Unquestionably, nutrition and food are thought to be important modifiable factors in the development of CRC. It is important to remember that 20–25% of cancer cases worldwide may be related to nutrition and diet [4]. Therefore, the incidence and impact of CRC can be significantly reduced by implementing primary prevention strategies, such as adopting a healthy lifestyle and diet, avoiding risk factors, and early detection through screening [5,6,7].

A diet–scope–association study from the European Prospective Investigation into Cancer and Nutrition (EPIC) showed that alcohol was positively associated with CRC risk, while dairy products and calcium were inversely associated with CRC risk. Moreover, they tested multiple gene–nutrient multiplication interactions, but none remained significant [8]. According to gene–lifestyle interactions, depending on a person’s genetic predisposition, changeable lifestyle factors may have varying effects on complex diseases [9]. Genome-wide association studies have found sites linked to CRC [10]. In addition, previous studies have shown that high intakes of processed red meat and alcohol are associated with increased CRC risk in the UK Biobank population, while cereal fiber is associated with a reduced risk of CRC [11]. However, such studies did not fully examine the relationship between a wide range of foods and nutrients and CRC, nor did they consider genetic–nutrition interactions. There are currently insufficient studies examining the relationship between various dietary components and genetic predisposition on the prevention of CRC in various large cohorts.

Dietary assessment data obtained from the UK Biobank 24 h online touchscreen diet assessment questionnaire have been demonstrated to be highly reproducible in earlier studies [12]. The purpose of this study was to systematically evaluate the relationship between the 139 foods and nutrients covered in the online questionnaire and the risk of CRC, and to explore relationship between genetic–nutrition interactions and CRC risk. We aimed to validate previous findings and to explore some new dietary factors associated with CRC risk, to complement the shortcomings of previous studies due to their inclusion of fewer food groups, combined with the results of large cohort studies in other populations, and thus to provide supportive evidence for the prevention of CRC at the dietary level.

## 2. Materials and Methods

### 2.1. Study Populations

The UK Biobank is a longitudinal large cohort study of 500,000 middle-aged people recruited between 2006 and 2010 from across the UK [13]. We used baseline information on the population collected by the UK Biobank Assessment Centre and information on cancer status identified through links to national registries. At the baseline assessment center, a touchscreen questionnaire containing information on socio-demographic characteristics, medical history, and lifestyle factors was issued and standardized anthropometric and biometric measurements were performed. On the touchscreen, all participants used signature capture devices for informed consent. All participants provided informed consent at recruitment, allowing for follow-up using data linkage to health records. The study was performed in accordance with the Declaration of Helsinki. Full details of the cohort study have been described elsewhere [13].

### 2.2. Assessment of Food and Nutrients Intake

Previous articles detailed dietary data collected by the UK Biobank [12]. In addition to the touchscreen questionnaire, the food nutrient data included in this paper were collected from the Oxford WebQ (a web-based, self-administered 24 h dietary questionnaire) on five separate occasions by participants recruited in 2009 and those who provided email addresses to the UK Biobank [14]. The 24 h dietary assessment questionnaire included nearly 200 questions related to dietary consumption and has been validated elsewhere using biomarkers [15,16]. Taking into account the effects of seasonal changes in diet, we calculated the average daily foods and nutrients’ intake to reduce measurement errors [15]. A total of 118,210 participants completed at least two 24 h online dietary assessments.

### 2.3. Outcome Ascertainment

The prevalence and incidence of cancer in the UK Biobank prospective cohort were determined through hospital episode records and the cancer and death registry. Eligible participants were enrolled from the date of reporting to an assessment center until the date of first registration of cancer, death, or last follow-up, whichever was earlier, except for non-melanoma skin cancer (ICD-10 C44). The endpoint we included in the analysis was the first diagnosis of CRC or the registry of CRC, whichever was diagnosed first. Colon cancer includes overlapping colon lesions (C18.8) and undefined lesions (C18.9). Cancers that occur in the rectosigmoid node (C19) and rectum (C20) are classified as rectal cancer.

### 2.4. Statistical Analysis

We examined the associations between CRC incidence and intake of 139 foods and nutrients recorded in the 24 h dietary questionnaire at recruitment. For the primary analysis, we excluded participants with baseline cancer and CRC (*n* = 34,963) from the UK Biobank cohort, of a total of 502,404 participants, further excluded participants with missing 24 h dietary questionnaire data and people who completed only one 24 h online questionnaire (*n* = 349,138), and those with implausible total energy intake (>25,104 or <2510.4 kJ/day in men, or >16,736 or <1673.6 kJ/day in women; *n* = 93) [17], and finally included 118,210 participants in the analysis of 139 dietary factors and CRC risk. Demographic characteristics are presented as medians with the interquartile range (IQR), or as numbers with corresponding percentages. The dietary records of participants who completed more than one 24 h online assessment were averaged for food or nutrient intake for analysis.

In the UK Biobank, we used a Cox proportional risk model to assess the association between 139 foods and nutrients and the risk of CRC, expressed as hazard ratio (HR) and 95% confidence interval (CI). Person-years were calculated from the time of enrollment to the date of CRC diagnosis, follow-up failure, death, or last follow-up, whichever occurred first. The model was adjusted for age at recruitment (continuous), sex(male/female), Townsend deprivation index (TDI, continuous), education (College/University degree or other), family history of CRC (yes/no/unknown), regular aspirin use (yes/no/unknown), bowel screening (yes/no/unknown), diabetes (yes/no/unknown), body mass index (BMI, kg/m^2^, continuous), physical activity (minutes per week, continuous), smoking (pack-years, continuous), and total energy intake (Kj/day, continuous) as potential confounding factors and was further stratified by sex and cancer site. False discovery rate (FDR) was adopted to adjust for multiple comparisons (*p*-values < 0.05) [18]. Food or nutrient intake was also analyzed as categorical variables by dividing into tertiles. The multivariate analysis was adjusted for the same covariates in the intake analysis of the 139 dietary factors described above. For the FDR-significant food and nutrients in the UK Biobank, the pairwise partial correlation coefficients were quantified using Spearman’s rho (r). In addition, we examined possible non-linear associations using a restricted cubic spline [19].

Based on the availability of imputed single nucleotide polymorphisms (SNPs), we created a polygenic risk score (PRS) for CRC. We calculated the PRS by summing the weight of each risk variant with the product of the number of risk alleles (0, 1, and 2) for all GWAS-recognized risk variants for all study participants. Details of the derivation of genetic risk scores have been published [20,21]. The PRS was categorized as either low, intermediate, or high, based on the tertile distribution of PRS among non-cases. For foods or nutrients with significant FDR in the UK biobank, we inferred the association between these dietary factors and CRC risk in different PRS groups. To test interactions between GRS and different dietary components for the outcomes, multiplicative interaction was assessed by adding a cross-product term into the model.

All analyses were performed using R version 4.1.0. We set statistical significance at a two-sided *p* value < 0.05.

## 3. Results

### 3.1. Study Characteristics

Characteristics of the overall study population are summarized in Table S1. After a mean follow-up of 12.8 years, we identified 1466 incidents of CRC among 118,210 UK participants. Of these, 842 were colon cancer and 359 were rectal cancer. The mean (SD) age of the 1466 CRC patients was 55.87 (7.83) years and almost 44.6% of the study population was male. Compared to the general population, CRC cases were more likely to be male and white, older, and less educated, and to have a higher TDI, more family history of bowel cancer, a high BMI, less physical activity, more smoking, and a higher prevalence of diabetes at baseline (Table S1).

### 3.2. Results in the UK Biobank

**Figure 1:**
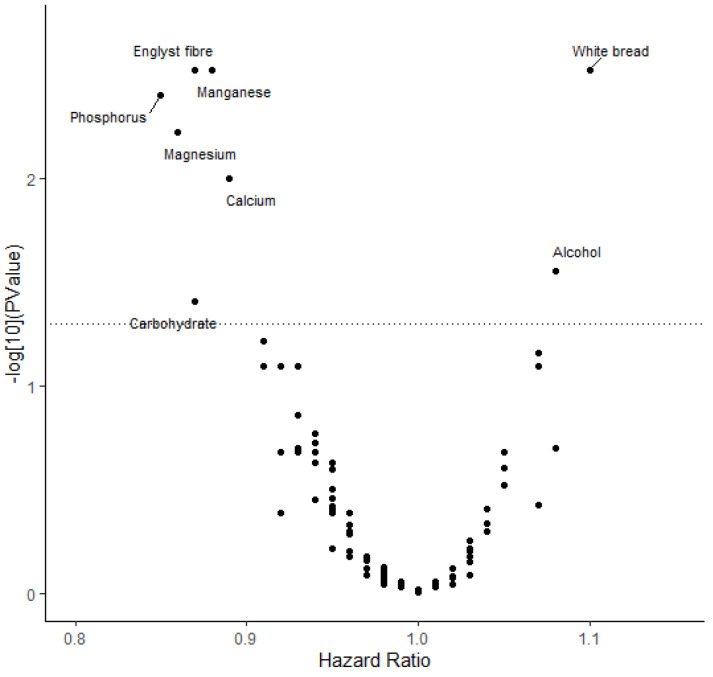
Volcano plot showing the association between 139 dietary factors and the risk of developing colorectal cancer in the UK Biobank. The *y*-axis shows the FDR-adjusted *p* values in −log10 scale from the Cox regression models for each dietary factor. The *x*-axis shows the estimated HR for each dietary factor per 1 SD increase per day. The dashed horizontal line represents the level of significance corresponding to FDR of 5%.

Of the 139 foods and nutrients that were examined in the UK Biobank, eight were associated with CRC risk (FDRP < 0.05) (Figure 1). The results showed that higher alcohol (HR per 1 SD increment in intake per day, 1.08; 95% CI: 1.03–1.14; FDRP = 0.028) and white bread (HR per 1 SD increment in intake per day, 1.10; 95% CI: 1.05–1.16; FDRP = 0.003) intake were associated with a higher risk of CRC, while dietary fibre, calcium, magnesium, phosphorus, manganese, and carbohydrate intake were associated with lower CRC risk, and the HRs for per 1 SD increment in intake per day were 0.87 (95% CI: 0.82–0.93; FDRP = 0.003), 0.89 (95% CI: 0.83–0.95; FDRP = 0.010), 0.86 (95% CI: 0.79–0.93; FDRP = 0.006), 0.85 (95% CI: 0.78–0.92; FDRP = 0.004), 0.88 (95% CI: 0.82–0.93; FDRP = 0.003), and 0.87 (95% CI: 0.79–0.95; FDRP = 0.039), respectively (Table S2). There were no significant associations of the remaining foods and nutrients with CRC risk (Table S2).

Table 1 shows the HRs and 95% CI for the eight dietary factors associated with CRC risk in a categorical variable analysis. There was a positive association between white bread and CRC risk (HR comparing highest to lowest tertile HR_T3vs.T1_ = 1.22; 95% CI: 1.08–1.37; *p* = 0.001). Levels of dietary fiber, calcium, magnesium, phosphorus, and manganese were inversely associated with CRC risk, and the HRs_T3vs.T1_ were 0.80 (95% CI: 0.69–0.93; *p* = 0.003), 0.80 (95% CI: 0.68–0.93; *p* = 0.003), 0.82 (95% CI: 0.69–0.97; *p* = 0.023), 0.81 (95% CI: 0.68–0.97; *p* = 0.020), and 0.76 (95% CI: 0.65–0.88; *p* = 2.19 × 10^−4^), respectively. Overall, all of the associations remained statistically significant after correcting for the FDR. In the subgroup analysis of cancer sites, high intake of white bread was associated with increased risk of both colon and rectal cancer, dietary calcium and manganese were associated with lower colon cancer risk, and dietary fiber and magnesium were associated with lower rectal cancer risk (Table 1).

In subgroup analyses that included dietary factors as continuity variables, the association of dietary factors in colon and rectal cancers varied significantly by tumor sublocation analysis. No dietary factors remained significant after multiple corrections were found in colon cancer. Dietary fiber and magnesium showed significant protective effects against CRC, and the HRs per 1 SD increment in intake per day were 0.77 (95% CI: 0.67–0.88; FDR = 0.018) and 0.74 (95% CI: 0.63–0.88; FDR = 0.031). Moreover, these two factors showed low heterogeneity among different tumor subtypes (P heterogeneity: 0.015, 0.072) (Table S3). In the sex-stratified analysis, the relationship between 139 dietary factors and CRC was largely different in the different sex populations. Among women, no dietary factor was significantly associated with CRC risk after multiple corrections (Table S4). However, in the male population, the same and greater results were observed than in the general population. In men, the protective factors for CRC incident are carbohydrate, dietary fiber, calcium, magnesium, phosphorus, and manganese (Table S4), while higher intake of alcohol, white bread, and processed meat are risk factors for CRC. Moderate inter-sex heterogeneity was observed for calcium, phosphorus, and alcohol (P heterogeneity: 0.326, 0.415, 0.415), while high inter-sex heterogeneity was observed for magnesium and white bread (P heterogeneity: 0.579, 0.582) (Table S4).

### 3.3. Sensitivity Analysis

**Figure 2:**
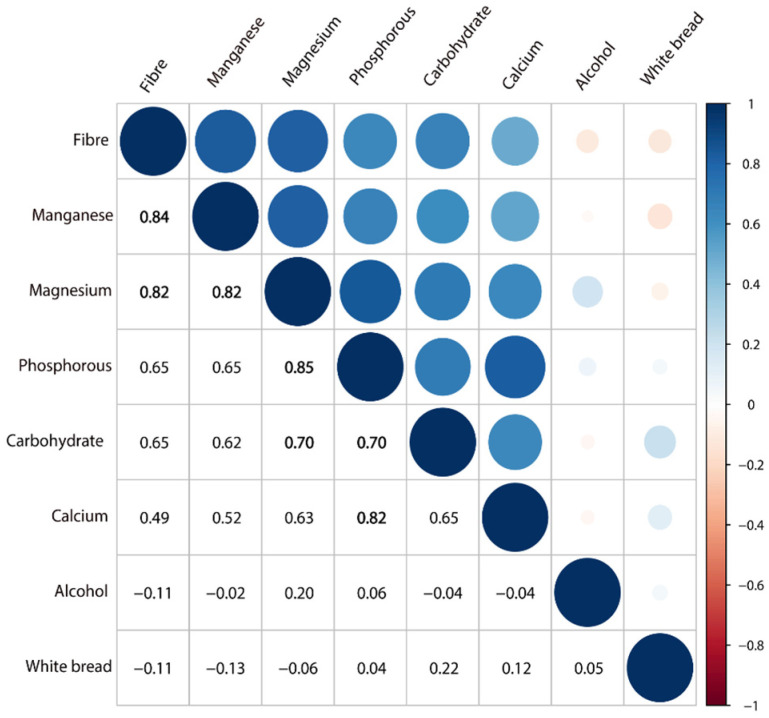
Pairwise partial correlation coefficients (Spearman’s r) of the eight FDR-significant foods and nutrients in the UK Biobank.

Pairwise correlations for the eight FDR-significant foods and nutrients are displayed in Figure 2. Dietary fiber intake was strongly correlated with intakes of magnesium (0.82) and manganese (0.84). Magnesium intake was strongly correlated with intakes of manganese (0.82) and phosphorus (0.85). Phosphorus intake was strongly correlated with intake of calcium (0.82). There were also notable correlations between intakes of carbohydrates and magnesium (0.70), and between carbohydrates and phosphorus (0.70). There was evidence of a non-linear relationship between alcohol (*p* = 0.007), magnesium (*p* = 0.006), and phosphorus (*p* = 0.018) intake and CRC risk (Figures S2, S5 and S7).

### 3.4. Interaction of Dietary Factors and Genetic Predisposition in CRC Risk

With the increase in genetic risk, the incidence rate and HR of CRC gradually increased (Table S5). The multivariate-adjusted model results showed that the HRs of the high- and intermediate-genetic-risk group were 2.55 (95% CI, 2.21–2.93) and 1.61 (95% CI, 1.39–1.87) compared with the low-genetic-risk group, and the HRs per SD of PRS increase was 1.54 (95% CI, 1.46–1.62) (Table S2). Further stratified analysis by genetic risk category showed that the association between carbohydrate (HR = 0.87, 95% CI, 0.76–0.99), calcium (HR = 0.89, 95% CI, 0.81–0.98), phosphorus (HR = 0.88, 95% CI, 0.78–1.00), alcohol (HR = 1.10, 95% CI, 1.02–1.18) and white bread (HR = 1.10, 95% CI, 1.03–1.18) intake and CRC was significant in the high-genetic-risk group (Table 2). However, no significant interaction between PRS–nutrient and CRC risk was found among the eight dietary factors.

## 4. Discussion

We systematically evaluated the associations between 139 dietary factors and the risk of CRC in a large prospective cohort study. Our study found that higher intakes of dietary fiber, magnesium, phosphorus, and manganese were associated with a lower risk of CRC, and alcohol drinking and higher intakes of white bread were associated with higher CRC risk. However, there was no significant interaction between PRS and these dietary factors in relation to the susceptibility of CRC risk.

Our study confirmed the previously reported positive association between alcohol and CRC risk [8,22,23,24]. Ethanol in any type of alcoholic beverage is a known risk factor for CRC because its first metabolite, acetaldehyde, has been evaluated as a human carcinogen by the International Agency for Research [3]. Ingested alcohol reaches colon cells and diffuses within the lumen, where ethanol is metabolized into acetaldehyde by microbial alcohol dehydrogenase, causing mucosal damage and the proliferation of regenerative cells [25]. Our previous study reaffirmed the dose-dependent association between alcohol intake and CRC risk, found that genetic predisposition to alcohol drinking would increase CRC risk, and revealed that the pathogenic effect of alcohol could be partly attributed to DNA methylation via regulating the expression of COLCA1/COLCA2 gene [24]. Although the results of the study showed that alcohol was positively associated with CRC risk in the high GRS group, there was no significant interaction between gene–alcohol and CRC risk.

Our study identified several minerals that were significantly associated with CRC risk. Results from the UK Biobank showed that high dietary intake of calcium, magnesium, and phosphorus were associated with lower CRC risk. These findings are consistent with findings in the EPIC cohort. The metabolic balance of calcium, magnesium, and phosphorus are related in many ways, and because the food sources of these minerals are relatively similar, it is difficult to distinguish their independent roles [26,27]. The third World Cancer Research Fund (WCRF) expert report strongly suggests that consumption of calcium reduces the risk of CRC [28], also confirmed in the large cohort study EPIC [8]. Although our results found an inverse association between calcium intake and CRC in both the intermediate- and high-GRS groups, no significant interaction was found between gene–calcium and CRC risk. The protective effect of phosphorus and magnesium intake on CRC has been demonstrated in previous cohorts [8], although no significant protective effect of serum phosphorus and magnesium levels against CRC has been observed at the genetic level [29]. The metabolism of calcium and phosphorus is related in many ways [26,27]. Both calcium and phosphorus are involved in bone calcification and decalcification [26]. Because several of these nutrients have a common intake source, a large correlation was observed between calcium and magnesium in the UK Biobank, and between magnesium and phosphorus, making it challenging to distinguish their independent roles.

Our study also found that the protective effect between manganese intake and CRC was robust enough after multiple corrections. There are few observational studies on manganese and CRC. The results of the case-control study found that manganese intake in CRC cases was lower than that in the control group, but no significant inverse association was found [30]. Animal experiments showed that tumor growth and metastasis were significantly enhanced in mice with manganese deficiency and tumor infiltration of CD8+ T cells was significantly reduced [31]. Manganese plays an important role in the anti-tumor immune response of cGAS-STING, which can improve the efficacy of clinical immunotherapy [31] and is involved in some enzymes, such as pyruvate carboxylase and arginase in mitochondria. Further cohort studies are needed to verify the relationship between manganese intake and CRC risk.

Our observational results suggest that dietary fiber is a protective factor for CRC, which is consistent with previous findings [8,32]. Dietary fiber can accelerate intestinal motility, dilute carcinogens in the colon, and ferment fiber into short-chain fatty acids by gut bacteria, all mechanisms that suggest dietary fiber intake may reduce the risk of malignancy and improve colon health [33,34]. In addition, dietary fiber can be used as a broad pillar of CRC prevention and adjuvant therapy [35]. Although there is a strong mechanism association between fiber intake and CRC risk, epidemiological studies have shown different outcomes between fiber and CRC from different food sources [34]. In a meta-analysis of prospective observational studies, fruit, vegetable, and legume fiber was not found to be associated with CRC, while cereal fiber was associated with reduced CRC risk [34]. Whole grains, a major source of cereal fiber, are inversely associated with CRC morbidity and mortality [34,36]. Consistent with previous studies, our results found that high white bread intake was a risk factor for CRC. Notably, whole grains are a major source of many vitamins, minerals, and phytochemicals that have anti-cancer properties and may influence CRC risk through several potential mechanisms [34].

There are many advantages in our study. Firstly, the study had a large population, a large number of CRC cases, a long follow-up period, and a wide range of confounding factors. Secondly, the researchers systematically assessed the association of a relatively comprehensive set of foods and nutrients with CRC risk, and, to reduce information errors, only people who completed at least two questionnaires were included in this study. Thirdly, the results were reported in this study considering multiple corrections and stratified analysis based on different anatomical subsites and gender. Finally, the PRS score was constructed to evaluate the relationship between dietary components and CRC risk through genetic risk stratification and to comprehensively evaluate the interaction between gene–dietary components and CRC risk. The main limitations of this study are that the analysis was limited to the European population and the extrapolation of the results was limited. In addition, there was no mutual adjustment of dietary exposures and, since many dietary items had a common source of intake, a strong association was found between several nutrients associated with CRC risk in the UK Biobank, making it difficult to assess their independent effects.

## 5. Conclusions

We systematically evaluated the association between dietary intakes of 139 foods and nutrients and the risk of CRC in the UK Biobank. Our study confirms the previously described positive association between alcohol and CRC risk. We additionally found that dietary intake of calcium, magnesium, phosphorus, manganese, and fiber was associated with reduced CRC risk, whilst white bread intake was associated with increased CRC risk, regardless of genetic background. Our study provides evidence and suggestions for dietary prevention of CRC. More and larger cohort studies are needed in the future to validate our results and explore more associations between dietary components and CRC risk.

## Figures and Tables

**Table 1:** The multivariable HR and 95% CI of colorectal cancer and its subsites according to tertiles of 8 foods and nutrients.

Foods and Nutrients	Colorectal Cancer				Colon Cancer			Rectal Cancer		
		Cases/Controls	Multivariable HR (95% CI)	*p* Value	Cases/Controls	Multivariable HR (95% CI)	*p* Value	Cases/Controls	Multivariable HR (95% CI)	*p* Value
Carbohydrate	T1	452/38,953	Ref.		269/39,015	Ref.		104/39,019	Ref.	
	T2	508/38,894	1.00 (0.87, 1.14)	0.966	279/39,004	0.95 (0.79, 1.14)	0.559	128/38,995	1.07 (0.81, 1.42)	0.628
	T3	506/38,897	0.84 (0.70, 1.01)	0.064	294/38,990	0.89 (0.70, 1.12)	0.317	127/38,995	0.87 (0.61, 1.26)	0.472
Dietary fiber	T1	468/38,936	Ref.		252/39,032	Ref.		123/39,000	Ref.	
	T2	521/38,882	0.98 (0.86, 1.12)	0.769	306/38,977	1.10 (0.92, 1.30)	0.295	129/38,993	0.91 (0.70, 1.17)	0.463
	T3	477/38,926	0.80 (0.69, 0.93)	0.003	284/39,000	0.94 (0.78, 1.15)	0.557	107/39,016	0.64 (0.48, 0.87)	0.004
Calcium	T1	478/38,926	Ref.		280/39,004	Ref.		113/39,010	Ref.	
	T2	504/38,900	0.93 (0.82, 1.06)	0.305	296/38,987	0.94 (0.79, 1.11)	0.465	125/38,997	0.98 (0.75, 1.28)	0.874
	T3	484/38,918	0.80 (0.68, 0.93)	0.003	266/39,018	0.75 (0.62, 0.92)	0.006	121/39,002	0.84 (0.62, 1.14)	0.261
Magnesium	T1	448/38,956	Ref.		259/39,025	Ref.		117/39,006	Ref.	
	T2	513/38,890	0.98 (0.85, 1.12)	0.727	295/38,988	0.99 (0.83, 1.18)	0.912	118/39,004	0.83 (0.63, 1.09)	0.172
	T3	505/38,898	0.82 (0.69, 0.97)	0.023	288/38,996	0.85 (0.68, 1.07)	0.167	124/38,999	0.70 (0.50, 0.98)	0.038
Phosphorus	T1	445/38,959	Ref.		257/39,027	Ref.		108/39,015	Ref.	
	T2	521/38,882	1.00 (0.88, 1.15)	0.946	297/38,986	1.01 (0.85, 1.21)	0.892	131/38,991	1.02 (0.78, 1.34)	0.875
	T3	500/38,903	0.81 (0.68, 0.97)	0.020	288/38,996	0.86 (0.68, 1.08)	0.195	120/39,003	0.76 (0.54, 1.09)	0.134
Manganese	T1	492/38,912	Ref.		289/38,995	Ref.		119/39,004	Ref.	
	T2	510/38,893	0.92 (0.81, 1.05)	0.233	288/38,995	0.90 (0.76, 1.06)	0.210	121/39,001	0.91 (0.70, 1.19)	0.494
	T3	464/38,939	0.76 (0.65, 0.88)	2.19E-04	265/39,019	0.76 (0.63, 0.92)	0.005	119/39,004	0.80 (0.59, 1.07)	0.128
Alcohol	T1	465/38,946	Ref.		274/39,011	Ref.		102/39,024	Ref.	
	T2	454/38,942	0.94 (0.83, 1.08)	0.385	265/39,017	0.94 (0.79, 1.11)	0.457	114/39,005	1.06 (0.81, 1.39)	0.654
	T3	547/38,856	1.02 (0.90, 1.17)	0.715	303/38,981	0.97 (0.82, 1.15)	0.731	143/38,980	1.17 (0.90, 1.52)	0.254
White bread	T1	625/53789	Ref.		356/53,912	Ref.		146/53,912	Ref.	
	T2	278/24,461	1.03 (0.89, 1.18)	0.716	167/24,508	1.08 (0.90, 1.30)	0.402	64/24,508	1.03 (0.77, 1.38)	0.845
	T3	563/38,494	1.22 (1.08, 1.37)	0.001	319/38,589	1.22 (1.05, 1.43)	0.012	149/38,589	1.35 (1.06, 1.70)	0.013

CI, confidence interval; HR, hazard ratio. All dietary factors were entered into the model as tripartite categorical variables. The model was adjusted for age at recruitment (continuous), sex(male/female), Townsend deprivation index (TDI, continuous), education (College or University degree/other), ethnicity(white/others), family history of CRC (yes/no/unknown)), regular aspirin use (yes/no/unknown), bowel screening(yes/no/unknown), diabetes(yes/no/unknown), body mass index (BMI, kg/m^2^, continuous), physical activity (minutes per week, continuous), smoking (pack-years, continuous), and total energy intake (kJ/day, continuous).

**Table 2:** The association of the eight foods and nutrients intake with colorectal cancer risk by genetic risk score stratification.

Dietary Factors	HR (95% CI)	*p* Value	*p* for Interaction
Carbohydrate			0.063
Low	0.93 (0.75, 1.15)	0.488	
Intermediate	0.85 (0.72, 1.00)	0.054	
High	0.87 (0.76, 0.99)	0.035	
Dietary fiber			0.627
Low	0.81 (0.70, 0.95)	0.008	
Intermediate	0.83 (0.74, 0.93)	0.002	
High	0.93 (0.85, 1.02)	0.139	
Calcium			0.295
Low	0.89 (0.76, 1.04)	0.155	
Intermediate	0.86 (0.76, 0.97)	0.017	
High	0.89 (0.81, 0.98)	0.022	
Magnesium			0.310
Low	0.80 (0.66, 0.97)	0.022	
Intermediate	0.84 (0.72, 0.97)	0.018	
High	0.89 (0.79, 1.00)	0.060	
Phosphorus			0.291
Low	0.79 (0.65, 0.96)	0.019	
Intermediate	0.82 (0.70, 0.96)	0.014	
High	0.88 (0.78, 1.00)	0.045	
Manganese			0.246
Low	0.83 (0.71, 0.97)	0.016	
Intermediate	0.86 (0.76, 0.97)	0.011	
High	0.91 (0.83, 1.00)	0.052	
Alcohol			0.779
Low	1.07 (0.96, 1.20)	0.225	
Intermediate	1.06 (0.97, 1.16)	0.170	
High	1.10 (1.02, 1.18)	0.011	
White bread			0.762
Low	1.18 (1.07, 1.31)	0.001	
Intermediate	1.07 (0.99, 1.17)	0.103	
High	1.10 (1.03, 1.18)	0.008	

HR hazard ratio; CI confidence interval. All dietary factors entered the models as standardized continuous variables and reflect associations per 1 SD increase in daily consumption. The model was adjusted for age at recruitment (continuous), sex(male/female), Townsend deprivation index (TDI, continuous), education (College or University degree/other), ethnicity(white/others), family history of CRC (yes/no/unknown)), regular aspirin use (yes/no/unknown), bowel screening(yes/no/unknown), diabetes(yes/no/unknown), body mass index (BMI, kg/m^2^, continuous), physical activity (minutes per week, continuous), smoking (pack-years, continuous), and total energy intake (kJ/day, continuous).

## Data Availability

Data described in the manuscript, code book, and analytic code will be made available upon request pending application and approval.

## Supplementary Materials

The following supporting information can be downloaded at: https://www.mdpi.com/article/10.3390/nu15224801/s1, Figure S1: Flow chart of study participants. Figure S2: Non-linear relationship between carbohydrate intake and colorectal cancer. Figure S3: Non-linear relationship between alcohol intake and colorectal cancer. Figure S4: Non-linear relationship between calcium intake and colorectal cancer. Figure S5: Non-linear relationship between dietary fibre intake and colorectal cancer. Figure S6: Non-linear relationship between magnesium intake and colorectal cancer. Figure S7: Non-linear relationship between manganese intake and colorectal cancer. Figure S8: Non-linear relationship between phosphorus intake and colorectal cancer. Figure S9: Non-linear relationship between white bread intake and colorectal cancer. Table S1: Baseline characteristics of study population. Table S2: The Association of 139 Foods and Nutrients Intake in Relation to Colorectal Cancer Risk in the UK Biobank. Table S3: The association of the 139 foods and nutrient intakes with colorectal cancer risk by tumor location (colon vs rectal). Table S4: The association of the 139 foods and nutrient intakes with colorectal cancer risk by gender stratification. Table S5: Risk of incident colorectal cancer according to genetic risk.
