# Supplementary material for: Diet-Wide Association, Genetic Susceptibility and Colorectal Cancer Risk: A Prospective Cohort Study
Source: Nutrients. 2023 Nov 16;15(22):4801. doi: 10.3390/nu15224801 (PMC10674290; doi:10.3390/nu15224801)
Supplement: Supplementary file 1 [file nutrients-15-04801-s001.zip › nutrients-2661364-supplementary.pdf]

On-line Supplementary Material

Diet-wide association, genetic susceptibility and colorectal cancer risk: a prospective cohort study

Jin et al.

Figure S1 Flow chart of study participants.

Figure S2 Non-linear relationship between carbohydrate intake and colorectal cancer.

Figure S3 Non-linear relationship between alcohol intake and colorectal cancer.

Figure S4 Non-linear relationship between calcium intake and colorectal cancer.

Figure S5 Non-linear relationship between dietary fibre intake and colorectal cancer.

Figure S6 Non-linear relationship between magnesium intake and colorectal cancer.

Figure S7 Non-linear relationship between manganese intake and colorectal cancer.

Figure S8 Non-linear relationship between phosphorus intake and colorectal cancer.

Figure S9 Non-linear relationship between white bread intake and colorectal cancer.

TableS1 Baseline characteristics of study population.

TableS2 The Association of 139 Foods and Nutrients Intake in Relation to Colorectal Cancer Risk in the UK Biobank.

TableS3 The association of the 139 foods and nutrient intakes with colorectal cancer risk by tumor location (colon vs rectal).

TableS4 The association of the 139 foods and nutrient intakes with colorectal cancer risk by gender stratification.

Table S5 Risk of incident colorectal cancer according to genetic risk.

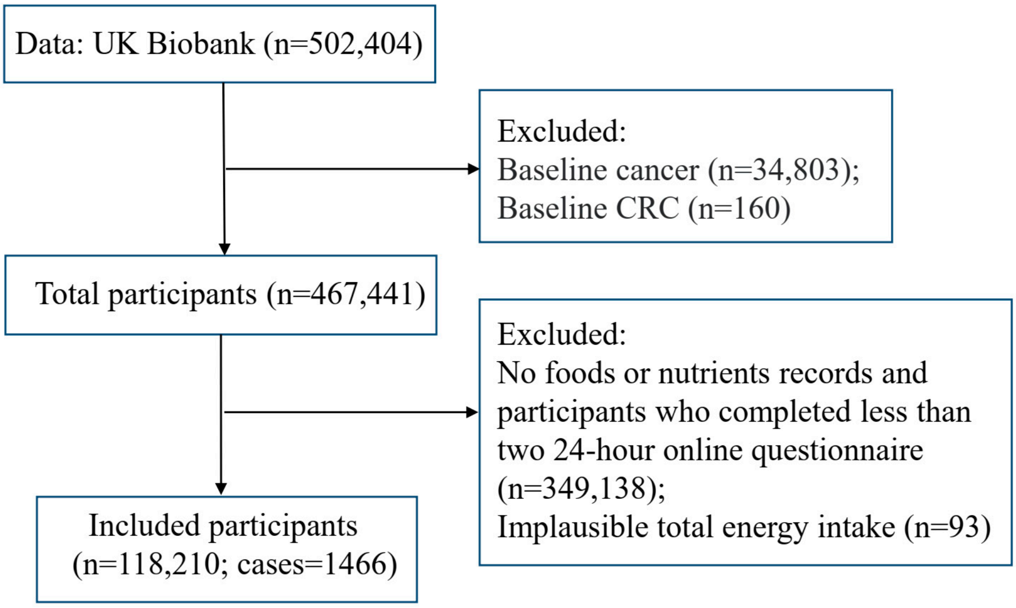

Figure S1 Flow chart of study participants.
CRC, colorectal cancer

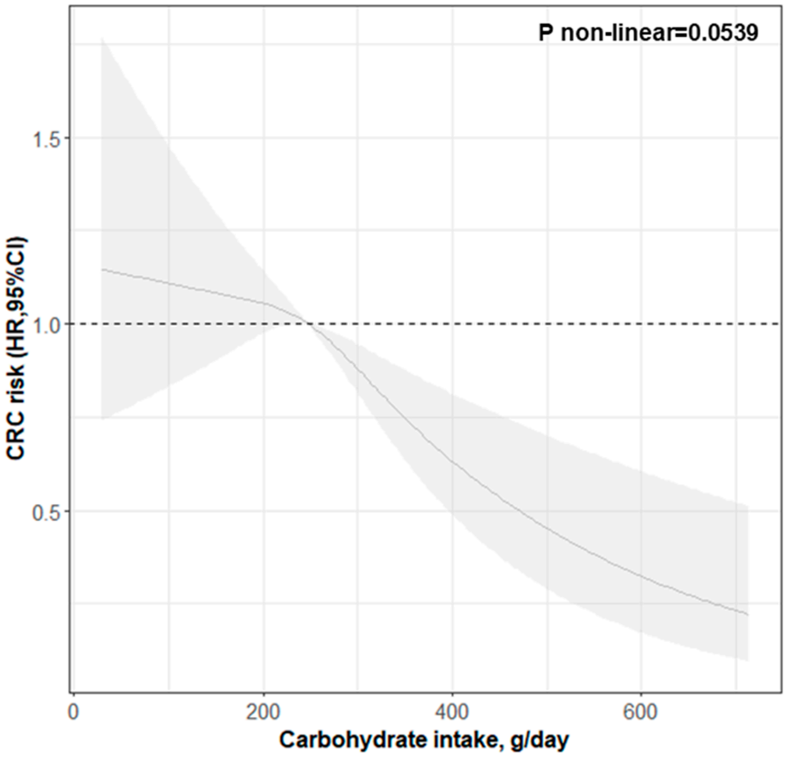

Figure S2 Non-linear relationship between carbohydrate intake and colorectal cancer
CI, confidence interval; HR, hazard ratio

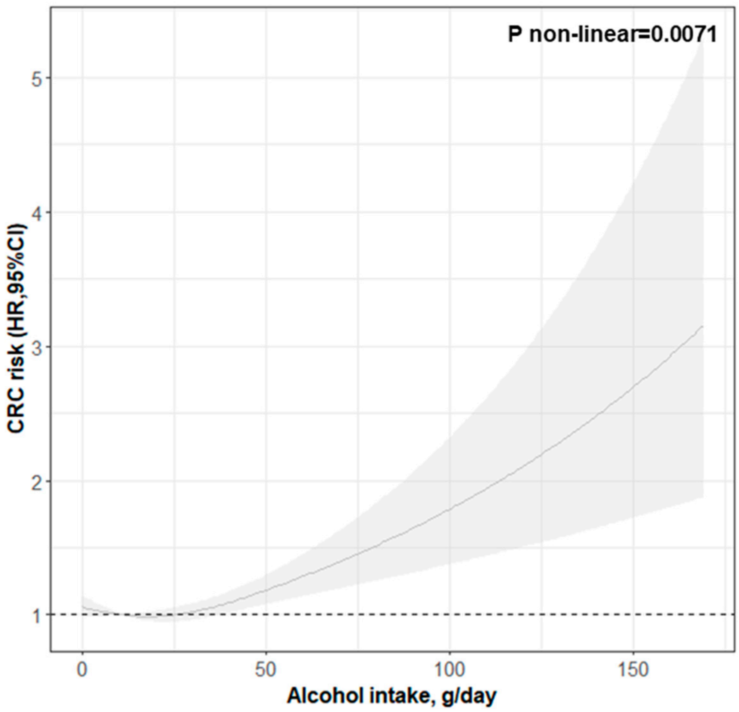

Figure S3 Non-linear relationship between alcohol intake and colorectal cancer
CI, confidence interval; HR, hazard ratio

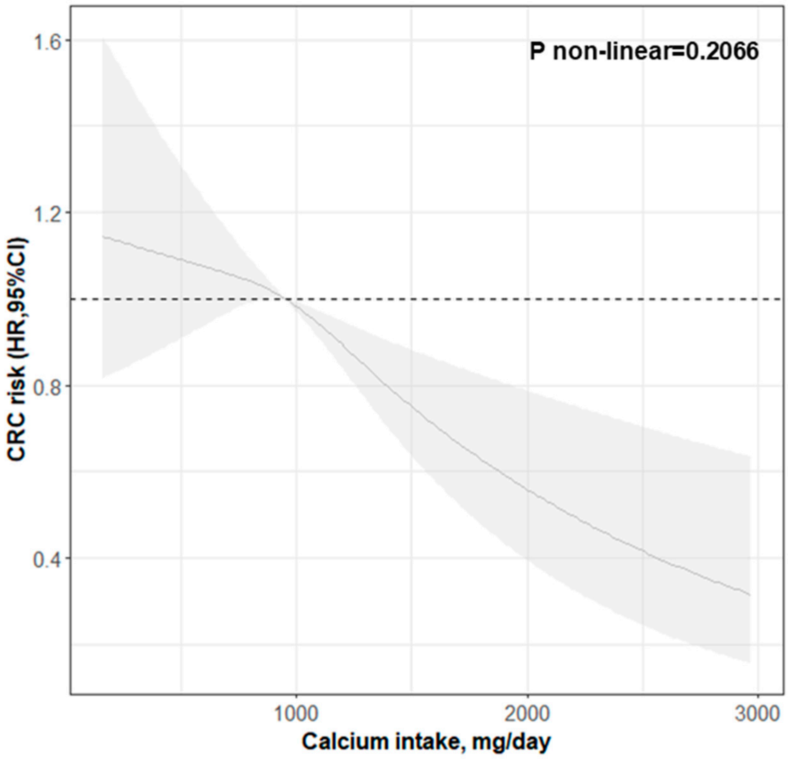

Figure S4 Non-linear relationship between calcium intake and colorectal cancer
CI, confidence interval; HR, hazard ratio

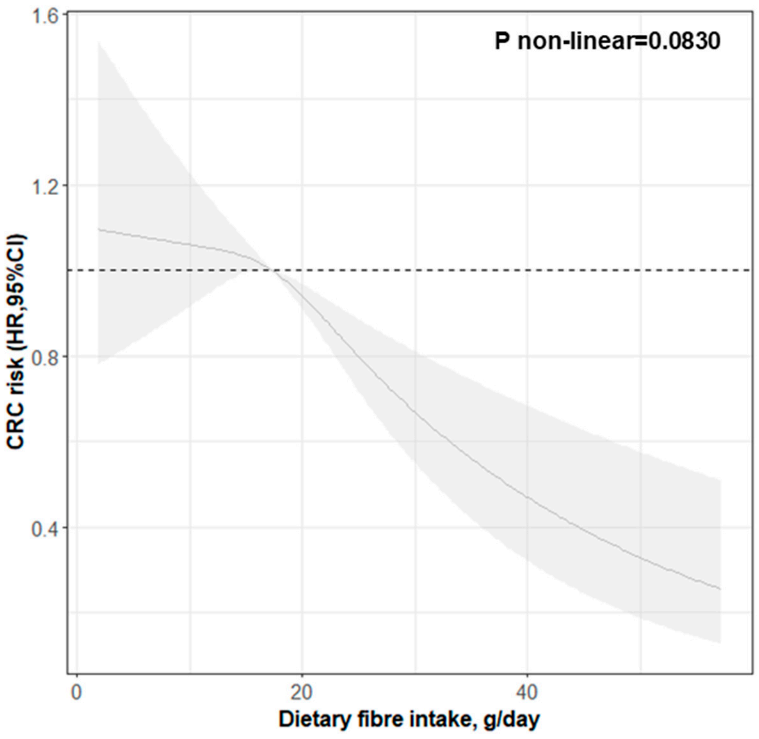

Figure S5 Non-linear relationship between dietary fibre intake and colorectal cancer
CI, confidence interval; HR, hazard ratio

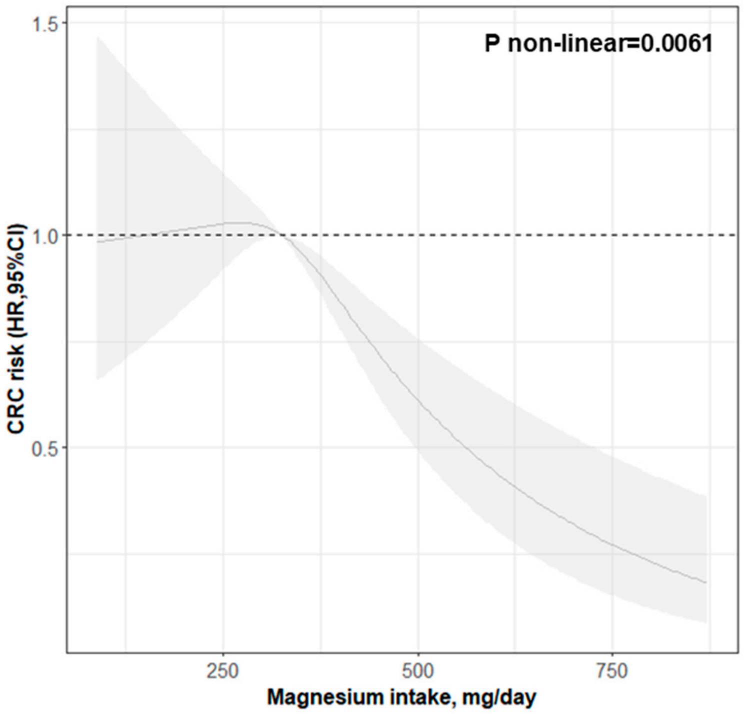

Figure S6 Non-linear relationship between magnesium intake and colorectal cancer
CI, confidence interval; HR, hazard ratio

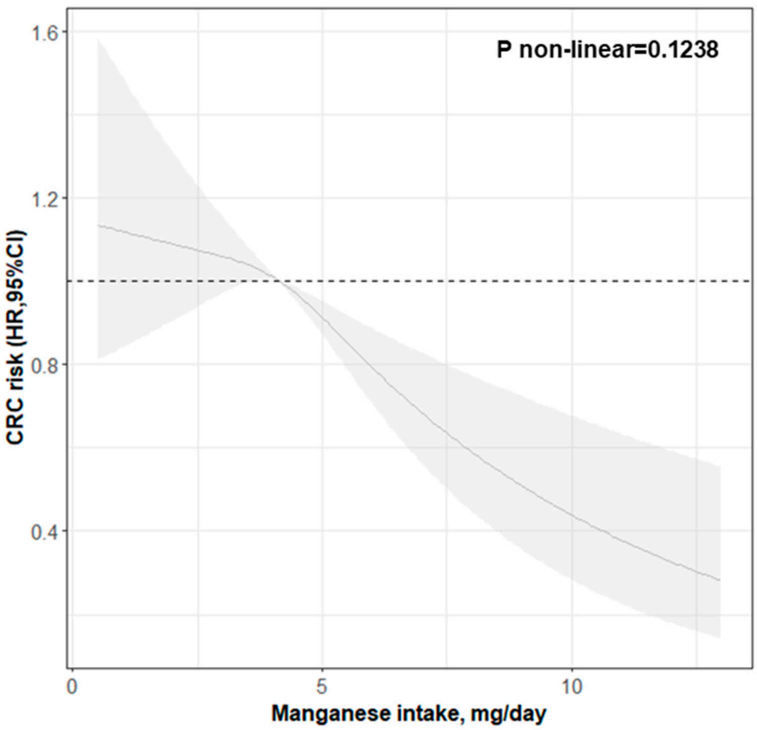

Figure S7 Non-linear relationship between manganese intake and colorectal cancer
CI, confidence interval; HR, hazard ratio

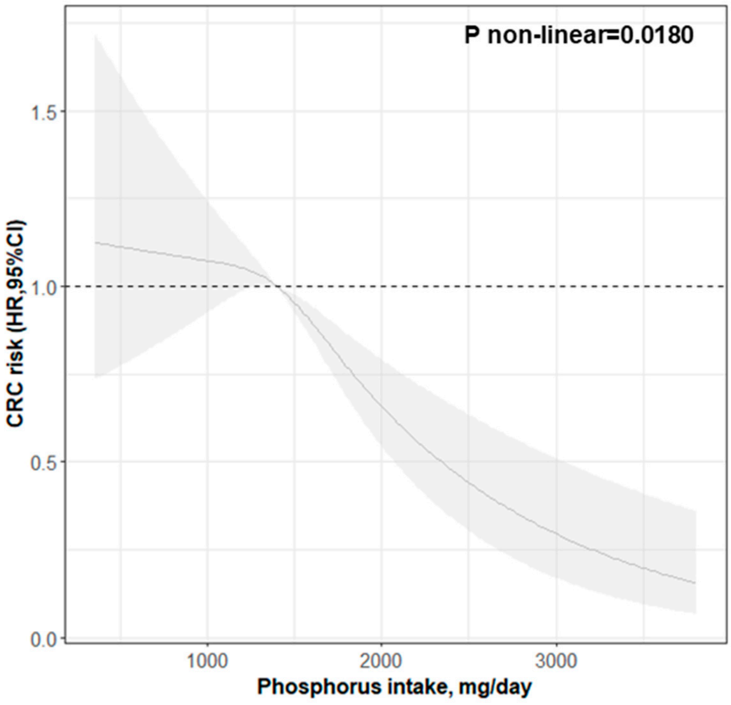

Figure S8 Non-linear relationship between phosphorus intake and colorectal cancer
CI, confidence interval; HR, hazard ratio

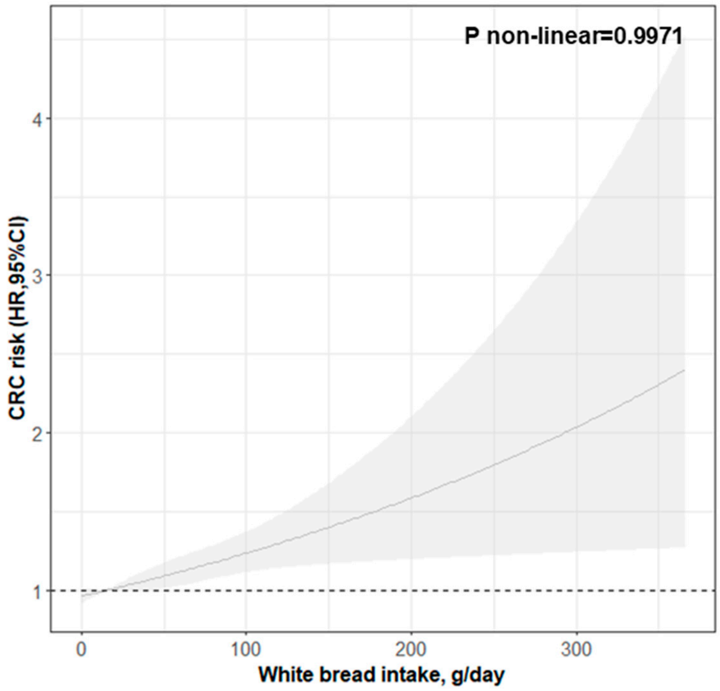

Figure S9 Non-linear relationship between white bread intake and colorectal cancer

CI, confidence interval; HR, hazard ratio

TableS1 Baseline characteristics of study population[1]

Characteristic	Overall<br>118,210	No incident CRC<br>116,744	Incident CRC<br>1,466
**Age, mean (SD)**	55.87 (7.83)	55.82 (7.83)	59.96 (6.71)
**Male (%)**	52,664 (44.6)	51,833 (44.4)	831 (56.7)
**TDI, mean (SD)**	-1.62 (2.84)	-1.62 (2.84)	-1.73 (2.88)
**College/University degree (%)**	55,588 (47.0)	54,930 (47.1)	658 (44.9)
**Ethnicity (%)**			
White	106,238 (89.9)	104,912 (89.9)	1,326 (90.5)
Others	119,72(10.1)	118,32(10.1)	140(0.5)
**Family history of bowel cancer (%)**			
No	85,258 (72.1)	84,270 (72.2)	988 (67.4)
Yes	7,920 (6.7)	7,779 (6.7)	141 (9.6)
Unknown	25,032 (21.2)	24,695 (21.2)	337 (23.0)
**Regular aspirin use, n (%)**			
No	103,445 (87.5)	102,225 (87.6)	1,220 (83.2)
Yes	13,877 (11.7)	13,641 (11.7)	236 (16.1)
Unknown	888 (0.8)	878 (0.8)	10 (0.7)
**Ever received bowel screening[2]**			
No	78,282 (66.2)	77,442 (66.3)	840 (57.3)
Yes	38,288 (32.4)	37,684 (32.3)	604 (41.2)
Unknown	1,640 (1.4)	1,618 (1.4)	22 (1.5)
**Diabetes diagnosed by doctor (%)**			
No	113,565 (96.1)	112,197 (96.1)	1,368 (93.3)
Yes	4,460 (3.8)	4,362 (3.7)	98 (6.7)
Unknown	185 (0.2)	185 (0.2)	0 (0.0)
**Body mass index (Kg/m2)**	26.72 (4.58)	26.71 (4.58)	27.41 (4.58)
**Physical activity (minutes/week)**	96.03 (110.55)	96.07 (110.43)	92.78 (119.45)
**Pack years of smoking**	19.34 (8.93)	19.32 (8.86)	21.05 (12.87)

[1] Data are presented as means (± SD) or proportions (percentages).

[2] Bowel screening includes tests for blood in the stool/feces or a colonoscopy or a sigmoidoscopy.

TableS2. The Association of 139 Foods and Nutrients Intake in Relation to Colorectal Cancer Risk in the UK Biobank

Foods and nutrients	HR (95%CI)	P	FDRP
**Nutrients**			
Carbohydrate	0.87(0.79,0.95)	0.002	0.039
Dietary fibre	0.87(0.82,0.93)	0.000	0.003
Calcium	0.89(0.83,0.95)	0.000	0.010
Magnesium	0.86(0.79,0.93)	0.000	0.006
Alcohol	1.08(1.03,1.14)	0.001	0.028
Phosphorus	0.85(0.78,0.92)	0.000	0.004
Manganese	0.88(0.82,0.93)	0.000	0.003
Protein	0.98(0.91,1.06)	0.692	0.908
Vegetable protein	0.93(0.86,0.99)	0.033	0.210
Animal protein	1.02(0.96,1.08)	0.562	0.839
Fat	1.01(0.92,1.11)	0.815	0.938
Vegetable fat	0.98(0.91,1.05)	0.525	0.834
Animal fat	1.03(0.96,1.10)	0.443	0.817
Saturated fatty acids	1.02(0.94,1.10)	0.702	0.908
n-3 fatty acids	0.98(0.93,1.05)	0.594	0.870
n-6 fatty acids	0.97(0.91,1.04)	0.437	0.817
Iron	0.95(0.88,1.03)	0.224	0.610
Vitamin B6	0.99(0.93,1.05)	0.724	0.913
Vitamin B12	1.00(0.95,1.06)	0.906	0.965
Folate	0.95(0.89,1.01)	0.094	0.383
Vitamin C	0.98(0.93,1.03)	0.396	0.786
Potassium	0.92(0.86,0.99)	0.031	0.210
Retinol	1.05(1.00,1.09)	0.032	0.210
Total carotene	1.00(0.95,1.06)	0.899	0.965
Vitamin E	1.00(0.93,1.07)	0.892	0.965
Vitamin D	1.00(0.95,1.05)	0.934	0.976
Starch	0.99(0.92,1.07)	0.758	0.913
Monounsaturated fatty acids	1.03(0.95,1.13)	0.467	0.822
Zinc	0.97(0.90,1.04)	0.374	0.754
Thiamin	0.93(0.87,0.99)	0.027	0.200
Riboflavin	0.91(0.85,0.97)	0.004	0.061
Cholesterol	1.01(0.95,1.06)	0.818	0.938
Alpha-carotene	1.01(0.96,1.07)	0.640	0.899
Beta-carotene	1.00(0.95,1.06)	0.861	0.955
Beta cryptoxanthin	0.96(0.91,1.02)	0.235	0.627
Biotin	0.94(0.88,1.01)	0.081	0.352
Chloride	1.07(0.99,1.15)	0.089	0.375
Copper	0.98(0.91,1.05)	0.486	0.834
Iodine	0.92(0.87,0.98)	0.007	0.080

Sodium	1.08(1.01,1.16)	0.027	0.200
Niacin equivalent	1.01(0.94,1.09)	0.740	0.913
Pantothenic acid	0.91(0.85,0.98)	0.008	0.081
Selenium	0.97(0.91,1.03)	0.288	0.663
Total nitrogen	0.97(0.89,1.05)	0.427	0.812
Vitamin A retinol equivalents	1.05(1.00,1.10)	0.047	0.250
Trans fatty acids	1.00(0.94,1.06)	0.995	0.995
**Foods**			
White bread	1.10(1.05,1.16)	0.000	0.003
Animal fat spread lower fat	0.99(0.95,1.05)	0.830	0.938
Animal fat spread normal	1.00(0.95,1.05)	0.972	0.988
Added sugars and preserves	0.97(0.92,1.03)	0.319	0.692
Allium vegetables	0.98(0.93,1.03)	0.413	0.797
Beef	1.03(0.98,1.08)	0.245	0.630
Beer and cider	1.05(1.00,1.10)	0.063	0.302
Biscuits	1.00(0.95,1.06)	0.866	0.955
Breaded/battered chicken	1.02(0.97,1.07)	0.374	0.754
Breaded/battered fish	0.94(0.89,1.00)	0.042	0.236
Mixed bread (50/50), brown and seeded	0.99(0.94,1.04)	0.705	0.908
Other bread	0.98(0.93,1.04)	0.496	0.834
Whole meal bread	0.94(0.89,0.99)	0.020	0.169
Biscuit cereal	0.98(0.94,1.04)	0.548	0.837
Bran cereal	0.93(0.88,0.99)	0.015	0.139
Oat cereal (non sugar)	0.95(0.90,1.00)	0.042	0.236
Oat cereal (sugar)	0.97(0.92,1.03)	0.305	0.674
Other cereal (sugar)	1.03(0.98,1.08)	0.189	0.556
Chocolate confectionery	1.02(0.96,1.07)	0.511	0.834
Coffee, caffeinated	1.01(0.96,1.06)	0.806	0.938
Coffee, decaffeinated	0.95(0.90,1.00)	0.051	0.253
Savoury crackers	0.98(0.93,1.03)	0.354	0.743
Milk-dairy desserts	0.98(0.93,1.03)	0.487	0.834
Other desserts and cakes and pastries	0.98(0.93,1.04)	0.511	0.834
Soy desserts and yogurt	0.99(0.94,1.05)	0.739	0.913
Milk-based and powdered drinks	1.00(0.95,1.05)	0.953	0.981
Egg and egg dishes	0.98(0.93,1.03)	0.358	0.743
Apples and pears	0.93(0.88,0.98)	0.007	0.080
Berries	0.99(0.94,1.05)	0.771	0.916
Citrus	1.00(0.95,1.05)	0.907	0.965
Dried fruit	0.96(0.91,1.01)	0.112	0.411
Other fruit	0.94(0.89,0.99)	0.023	0.189
Stewed fruit	0.98(0.93,1.03)	0.458	0.817
Fruit juice	1.01(0.96,1.06)	0.823	0.938
Full fat yogurt	1.01(0.96,1.06)	0.667	0.908
Grain dishes - added fat	0.99(0.94,1.05)	0.829	0.938

Green leafy/cabbages	1.00(0.95,1.05)	0.993	0.995
High fat cheese	1.01(0.96,1.06)	0.712	0.908
Lamb	1.03(0.98,1.08)	0.239	0.627
Legumes and pulses	0.98(0.93,1.04)	0.527	0.834
Low fat yogurt	0.94(0.89,1.00)	0.035	0.210
Medium and low fat cheese	0.96(0.91,1.01)	0.141	0.467
Other meat, offal	0.98(0.93,1.04)	0.532	0.834
Muesli	0.98(0.93,1.04)	0.537	0.834
Nut-based spreads	1.03(0.98,1.08)	0.221	0.610
Unsalted nuts and seeds	0.95(0.90,1.01)	0.078	0.350
Salted nuts and seeds	1.00(0.96,1.06)	0.853	0.955
Oily fish	0.99(0.94,1.04)	0.708	0.908
Olive oil (drizzling/dunking)	0.99(0.94,1.04)	0.639	0.899
Plant-based spread lower fat	1.04(0.99,1.09)	0.100	0.395
Plant-based spread normal	1.04(0.99,1.09)	0.137	0.464
White pasta and rice	0.97(0.92,1.03)	0.301	0.674
Whole meal pasta, brown rice and other wholegrains	0.96(0.91,1.02)	0.164	0.506
Peas and sweetcorn	0.99(0.94,1.04)	0.762	0.913
Pizza	1.01(0.96,1.07)	0.693	0.908
Pork	0.99(0.94,1.04)	0.615	0.881
Potatoes and sweet potatoes (baked/boiled)	1.00(0.95,1.05)	0.909	0.965
Fried/roast potatoes	1.03(0.98,1.08)	0.330	0.706
Mashed potatoes	1.03(0.98,1.08)	0.284	0.663
Poultry	1.04(0.99,1.09)	0.159	0.506
Processed meat	1.07(1.02,1.12)	0.008	0.080
Raw salad	1.01(0.96,1.07)	0.681	0.908
Rice/oat milk	0.92(0.82,1.02)	0.118	0.411
Root vegetables	0.98(0.93,1.04)	0.564	0.839
Low/non sugar sugar-sweetened beverages	0.99(0.94,1.05)	0.753	0.913
Sugar-sweetened beverages and other sugary drinks	1.00(0.95,1.05)	0.946	0.981
Samosa, pakora	0.98(0.93,1.04)	0.540	0.834
Sauces and condiments (high fat)	1.05(1.00,1.11)	0.049	0.250
Sauces and condiments (low fat)	0.99(0.94,1.04)	0.677	0.908
Semi skimmed milk	0.97(0.92,1.02)	0.290	0.663
Shellfish	1.03(0.98,1.08)	0.291	0.663
Skimmed milk and cholesterol-lowering milk	0.95(0.90,1.00)	0.068	0.317
Savoury snacks	1.04(0.98,1.09)	0.162	0.506
Soups	1.04(0.99,1.09)	0.105	0.395
Soy milk	0.98(0.92,1.04)	0.457	0.817
Meat substitutes - soy	0.96(0.89,1.03)	0.288	0.663
Spirits	0.98(0.93,1.03)	0.409	0.797
Sushi	1.03(0.98,1.08)	0.192	0.556
Other sweets	0.98(0.93,1.04)	0.568	0.839
Tea	0.98(0.93,1.03)	0.451	0.817

Tea, decaffeinated	0.95(0.90,1.01)	0.118	0.411
Tomatoes	1.00(0.95,1.05)	0.974	0.988
Vegetable dips	0.98(0.93,1.04)	0.500	0.834
Meat substitutes - vegetarian	1.01(0.96,1.07)	0.614	0.881
Other vegetables	0.96(0.91,1.02)	0.171	0.517
Vegetable side dishes	0.95(0.90,1.01)	0.105	0.395
Water (still and sparkling)	1.03(0.98,1.09)	0.222	0.610
White fish and tinned tuna	0.97(0.92,1.02)	0.286	0.663
Whole milk	1.01(0.96,1.06)	0.747	0.913
Fortified wine	0.99(0.94,1.04)	0.668	0.908
Red wine	1.07(1.02,1.12)	0.005	0.070
White wine	1.03(0.98,1.08)	0.250	0.631
Cream	1.00(0.95,1.05)	0.926	0.975

CI, confidence interval; FDR, false discovery rate; HR, hazard ratio

All dietary factors entered the models as standardized continuous variables and reflect associations per 1-SD increase in daily consumption. The model was adjusted for age at recruitment (continuous), sex(male/female), Townsend deprivation index (TDI, continuous), education (College or University degree/other), ethnicity(white/others), family history of CRC (yes/no/unknown)), regular aspirin use (yes/no/unknown), bowel screening(yes/no/unknown), diabetes(yes/no/unknown), body mass index (BMI, kg/m2, continuous), physical activity (minutes per week, continuous), smoking (pack-years, continuous), and total energy intake (KJ/day, continuous).

Table S3 The Association of the 139 Food and Nutrient Intakes with Colorectal Cancer Risk by Tumor Location (Colon vs Rectal)

Foods and Nutrients	Colon			Rectal			P value for Heterogeneity
	HR (95%CI)	P	FDR	HR (95%CI)	P	FDR	
**Nutrients**							
Dietary fibre	0.94(0.86,1.02)	0.143	0.651	0.77(0.67,0.88)	0.000	0.018	0.015
Magnesium	0.89(0.80,1.00)	0.043	0.530	0.74(0.63,0.88)	0.000	0.031	0.072
Protein	0.99(0.90,1.10)	0.899	0.986	0.97(0.83,1.13)	0.677	0.889	0.828
Vegetable protein	0.96(0.87,1.05)	0.387	0.878	0.85(0.73,0.99)	0.032	0.224	0.183
Animal protein	1.01(0.93,1.10)	0.791	0.986	1.04(0.92,1.17)	0.565	0.845	0.696
Fat	0.99(0.87,1.13)	0.923	0.986	1.06(0.88,1.29)	0.535	0.836	0.563
Vegetable fat	1.00(0.91,1.10)	0.976	0.991	0.89(0.77,1.02)	0.091	0.325	0.178
Animal fat	0.99(0.91,1.09)	0.900	0.986	1.15(1.01,1.32)	0.042	0.236	0.069
Carbohydrate	0.91(0.80,1.03)	0.119	0.651	0.78(0.65,0.93)	0.006	0.101	0.168
Saturated fatty acids	0.99(0.89,1.10)	0.899	0.986	1.11(0.95,1.29)	0.199	0.544	0.228
n-3 fatty acids	0.98(0.90,1.06)	0.549	0.892	1.04(0.93,1.17)	0.490	0.836	0.409
n-6 fatty acids	0.99(0.91,1.09)	0.909	0.986	0.92(0.80,1.06)	0.267	0.617	0.390
Calcium	0.89(0.81,0.97)	0.008	0.287	0.87(0.76,1.00)	0.050	0.236	0.786
Iron	0.99(0.89,1.10)	0.791	0.986	0.85(0.73,1.00)	0.056	0.250	0.115
Vitamin B6	1.01(0.92,1.10)	0.901	0.986	0.96(0.84,1.09)	0.530	0.836	0.529
Vitamin B12	1.00(0.93,1.07)	0.991	0.991	1.03(0.92,1.14)	0.620	0.872	0.651
Folate	0.99(0.91,1.08)	0.816	0.986	0.88(0.77,1.00)	0.044	0.236	0.140
Vitamin C	1.01(0.94,1.09)	0.724	0.986	0.93(0.83,1.04)	0.183	0.526	0.231
Potassium	0.97(0.88,1.06)	0.474	0.890	0.81(0.70,0.95)	0.007	0.101	0.048
Retinol	1.07(1.01,1.12)	0.015	0.343	1.01(0.92,1.11)	0.766	0.909	0.291
Total carotene	1.05(0.98,1.12)	0.158	0.651	0.97(0.87,1.08)	0.554	0.836	0.222
Vitamin E	1.01(0.92,1.11)	0.837	0.986	1.00(0.87,1.16)	0.947	0.990	0.910
Vitamin D	0.96(0.90,1.04)	0.323	0.875	1.11(1.00,1.22)	0.042	0.236	0.021
Alcohol	1.06(0.99,1.13)	0.071	0.552	1.13(1.02,1.24)	0.013	0.154	0.288
Starch	0.97(0.88,1.08)	0.587	0.892	1.02(0.88,1.19)	0.787	0.909	0.589
Monounsaturated fatty acids	1.02(0.91,1.15)	0.753	0.986	1.01(0.85,1.21)	0.906	0.970	0.927
Zinc	0.96(0.87,1.06)	0.426	0.878	0.92(0.79,1.06)	0.239	0.586	0.638
Thiamin	0.98(0.90,1.06)	0.594	0.892	0.86(0.75,0.98)	0.026	0.193	0.103
Riboflavin	0.91(0.83,0.99)	0.026	0.516	0.88(0.77,1.00)	0.048	0.236	0.677
Phosphorus	0.85(0.76,0.95)	0.006	0.287	0.81(0.68,0.96)	0.016	0.154	0.646
Cholesterol	0.98(0.91,1.06)	0.635	0.901	1.09(0.98,1.21)	0.106	0.367	0.109
Alpha-carotene	1.06(0.99,1.13)	0.077	0.563	0.97(0.87,1.08)	0.554	0.836	0.170
Beta-carotene	1.05(0.98,1.12)	0.149	0.651	0.97(0.87,1.08)	0.580	0.846	0.222
Beta cryptoxanthin	0.98(0.90,1.05)	0.552	0.892	0.96(0.84,1.09)	0.495	0.836	0.790
Biotin	0.94(0.86,1.02)	0.140	0.651	0.92(0.81,1.05)	0.232	0.586	0.786
Chloride	1.05(0.95,1.16)	0.312	0.875	1.11(0.95,1.29)	0.173	0.523	0.551
Copper	1.03(0.94,1.12)	0.515	0.892	0.84(0.72,0.97)	0.021	0.169	0.021
Iodine	0.93(0.86,1.00)	0.055	0.530	0.96(0.86,1.08)	0.510	0.836	0.649

Manganese	0.89(0.82,0.97)	0.010	0.287	0.82(0.72,0.94)	0.003	0.098	0.308
Sodium	1.05(0.95,1.15)	0.341	0.875	1.15(1.00,1.33)	0.047	0.236	0.299
Niacin equivalent	1.04(0.94,1.15)	0.427	0.878	0.96(0.82,1.11)	0.547	0.836	0.388
Pantothenic acid	0.92(0.84,1.00)	0.060	0.530	0.87(0.76,1.00)	0.051	0.236	0.501
Selenium	0.96(0.89,1.04)	0.314	0.875	1.00(0.89,1.12)	0.996	1.000	0.564
Total nitrogen	0.97(0.87,1.08)	0.611	0.892	0.96(0.82,1.13)	0.621	0.872	0.916
Vitamin A retinol equivalents	1.08(1.03,1.14)	0.004	0.287	1.00(0.90,1.11)	1.000	1.000	0.195
**Foods**							
Animal fat spread lower fat	0.95(0.88,1.02)	0.144	0.651	1.04(0.96,1.14)	0.327	0.694	0.117
Animal fat spread normal	1.00(0.94,1.07)	0.934	0.986	1.05(0.96,1.16)	0.277	0.622	0.404
Added sugars and preserves	0.93(0.87,1.00)	0.063	0.530	1.01(0.92,1.12)	0.762	0.909	0.180
Allium vegetables	1.02(0.95,1.09)	0.613	0.892	0.99(0.89,1.10)	0.893	0.970	0.643
Beef	1.02(0.95,1.09)	0.607	0.892	1.02(0.92,1.13)	0.715	0.889	1.000
Beer and cider	1.00(0.94,1.08)	0.895	0.986	1.12(1.03,1.22)	0.006	0.101	0.043
Biscuits	0.97(0.90,1.04)	0.352	0.875	1.03(0.94,1.14)	0.527	0.836	0.329
Breaded/battered chicken	1.04(0.98,1.11)	0.210	0.749	1.00(0.90,1.11)	0.956	0.992	0.529
Breaded/battered fish	1.00(0.93,1.06)	0.890	0.986	0.93(0.83,1.05)	0.236	0.586	0.290
Mixed bread (50/50), brown and seeded	1.03(0.97,1.10)	0.317	0.875	0.89(0.80,1.00)	0.042	0.236	0.025
Other bread	0.97(0.90,1.04)	0.390	0.878	0.99(0.89,1.10)	0.811	0.917	0.755
White bread	1.09(1.02,1.16)	0.010	0.287	1.15(1.05,1.25)	0.003	0.098	0.332
Whole meal bread	0.94(0.87,1.00)	0.065	0.530	0.96(0.87,1.07)	0.472	0.836	0.741
Biscuit cereal	1.03(0.97,1.10)	0.367	0.878	0.90(0.81,1.01)	0.068	0.267	0.037
Bran cereal	0.95(0.88,1.02)	0.159	0.651	0.87(0.77,0.98)	0.026	0.193	0.223
Oat cereal (non sugar)	0.92(0.86,0.99)	0.032	0.530	0.97(0.87,1.07)	0.543	0.836	0.407
Oat cereal (sugar)	0.99(0.92,1.06)	0.784	0.986	0.89(0.77,1.02)	0.086	0.323	0.185
Other cereal (sugar)	1.01(0.94,1.08)	0.819	0.986	1.11(1.02,1.22)	0.019	0.162	0.102
Chocolate confectionery	1.06(0.99,1.14)	0.082	0.565	0.95(0.84,1.07)	0.373	0.729	0.125
Coffee, caffeinated	0.99(0.93,1.06)	0.775	0.986	0.98(0.89,1.09)	0.755	0.909	0.869
Coffee, decaffeinated	0.96(0.89,1.03)	0.232	0.749	0.87(0.77,0.99)	0.039	0.236	0.184
Savoury crackers	0.94(0.87,1.01)	0.099	0.627	0.98(0.88,1.10)	0.773	0.909	0.543
Milk-dairy desserts	0.95(0.88,1.01)	0.118	0.651	1.06(0.96,1.16)	0.238	0.586	0.067
Other desserts and cakes and pastries	0.98(0.91,1.05)	0.548	0.892	0.99(0.89,1.10)	0.882	0.967	0.876
Soy desserts and yogurt	1.00(0.93,1.07)	0.943	0.986	0.95(0.82,1.10)	0.491	0.836	0.537
Milk-based and powdered drinks	1.02(0.95,1.09)	0.594	0.892	0.92(0.82,1.04)	0.200	0.544	0.141
Egg and egg dishes	0.95(0.88,1.02)	0.149	0.651	1.06(0.96,1.17)	0.240	0.586	0.082
Apples and pears	0.96(0.90,1.03)	0.265	0.819	0.90(0.81,1.01)	0.067	0.267	0.328
Berries	1.01(0.94,1.08)	0.865	0.986	0.92(0.81,1.04)	0.185	0.526	0.201
Citrus	1.04(0.98,1.11)	0.239	0.755	0.91(0.81,1.01)	0.088	0.323	0.039
Dried fruit	1.00(0.94,1.07)	0.982	0.991	0.85(0.74,0.97)	0.018	0.162	0.034
Other fruit	0.98(0.91,1.05)	0.561	0.892	0.84(0.74,0.94)	0.004	0.098	0.030
Stewed fruit	1.00(0.94,1.07)	0.981	0.991	0.95(0.85,1.06)	0.365	0.726	0.432
Fruit juice	1.03(0.97,1.11)	0.330	0.875	0.95(0.85,1.06)	0.340	0.694	0.221
Full fat yogurt	1.02(0.95,1.09)	0.616	0.892	1.03(0.94,1.14)	0.512	0.836	0.872

Grain dishes - added fat	0.98(0.91,1.05)	0.495	0.890	1.02(0.92,1.12)	0.753	0.909	0.519
Green leafy/cabbages	0.99(0.93,1.06)	0.872	0.986	1.08(0.98,1.18)	0.122	0.397	0.133
High fat cheese	1.00(0.93,1.07)	0.984	0.991	1.05(0.94,1.16)	0.391	0.744	0.449
Lamb	1.03(0.97,1.09)	0.359	0.875	1.03(0.94,1.13)	0.550	0.836	1.000
Legumes and pulses	0.99(0.93,1.06)	0.866	0.986	0.94(0.84,1.05)	0.247	0.593	0.432
Low fat yogurt	0.98(0.92,1.05)	0.633	0.901	0.84(0.74,0.95)	0.006	0.101	0.033
Medium and low fat cheese	0.97(0.91,1.05)	0.455	0.878	0.87(0.75,1.00)	0.045	0.236	0.184
Other meat, offal	0.99(0.92,1.06)	0.676	0.950	0.87(0.75,1.01)	0.060	0.262	0.124
Muesli	0.97(0.91,1.04)	0.442	0.878	0.99(0.89,1.10)	0.798	0.909	0.749
Nut-based spreads	1.00(0.93,1.08)	0.960	0.991	1.02(0.93,1.13)	0.642	0.884	0.752
Unsalted nuts and seeds	0.97(0.90,1.04)	0.409	0.878	0.89(0.78,1.01)	0.069	0.267	0.255
Salted nuts and seeds	1.02(0.96,1.09)	0.500	0.890	0.98(0.88,1.09)	0.709	0.889	0.529
Oily fish	0.97(0.91,1.04)	0.453	0.878	1.05(0.95,1.15)	0.328	0.694	0.183
Olive oil (drizzling/dunking)	0.99(0.92,1.06)	0.687	0.955	0.98(0.87,1.09)	0.686	0.889	0.881
Plant-based spread lower fat	1.06(1.00,1.12)	0.061	0.530	0.96(0.87,1.06)	0.378	0.729	0.088
Plant-based spread normal	1.05(0.98,1.11)	0.159	0.651	1.09(1.00,1.18)	0.062	0.263	0.479
White pasta and rice	0.98(0.91,1.05)	0.570	0.892	1.00(0.90,1.12)	0.971	0.993	0.762
Whole meal pasta, brown rice and other wholegrains	0.99(0.92,1.06)	0.769	0.986	0.95(0.85,1.07)	0.434	0.804	0.550
Peas and sweetcorn	0.97(0.91,1.04)	0.446	0.878	1.12(1.02,1.23)	0.015	0.154	0.014
Pizza	1.01(0.94,1.09)	0.818	0.986	1.05(0.95,1.16)	0.335	0.694	0.540
Pork	0.97(0.91,1.04)	0.438	0.878	0.99(0.89,1.09)	0.838	0.939	0.742
Potatoes and sweet potatoes (baked/boiled)	0.96(0.89,1.02)	0.195	0.734	1.05(0.96,1.16)	0.271	0.617	0.132
Fried/roast potatoes	1.04(0.97,1.11)	0.290	0.875	0.99(0.89,1.10)	0.884	0.967	0.442
Mashed potatoes	1.03(0.96,1.09)	0.414	0.878	1.06(0.97,1.16)	0.180	0.526	0.608
Poultry	1.05(0.98,1.12)	0.142	0.651	1.00(0.90,1.11)	0.992	1.000	0.442
Processed meat	1.07(1.00,1.14)	0.038	0.530	1.12(1.02,1.23)	0.015	0.154	0.433
Raw salad	1.02(0.95,1.10)	0.529	0.892	0.97(0.87,1.09)	0.616	0.872	0.464
Rice/oat milk	0.89(0.75,1.06)	0.195	0.734	0.99(0.87,1.12)	0.866	0.963	0.330
Root vegetables	1.03(0.96,1.10)	0.417	0.878	0.95(0.85,1.06)	0.357	0.718	0.222
Low/non sugar sugar-sweetened beverages	0.98(0.91,1.06)	0.588	0.892	0.97(0.86,1.10)	0.670	0.889	0.890
Sugar-sweetened beverages and other sugary drinks	1.01(0.94,1.08)	0.858	0.986	1.01(0.90,1.12)	0.911	0.970	1.000
Samosa, pakora	0.97(0.90,1.05)	0.491	0.890	1.02(0.92,1.13)	0.717	0.889	0.443
Sauces and condiments (high fat)	1.04(0.97,1.11)	0.228	0.749	1.08(0.98,1.20)	0.123	0.397	0.543
Sauces and condiments (low fat)	1.00(0.93,1.07)	0.942	0.986	0.98(0.88,1.09)	0.707	0.889	0.757
Semi skimmed milk	0.96(0.89,1.03)	0.218	0.749	0.96(0.86,1.07)	0.449	0.821	1.000
Shellfish	1.04(0.99,1.11)	0.141	0.651	0.99(0.89,1.11)	0.922	0.970	0.438
Skimmed milk and cholesterol-lowering milk	0.96(0.89,1.03)	0.222	0.749	0.97(0.87,1.08)	0.537	0.836	0.876
Savoury snacks	1.03(0.95,1.10)	0.490	0.890	1.01(0.90,1.12)	0.920	0.970	0.770

Soups	1.03(0.97,1.10)	0.353	0.875	0.95(0.85,1.06)	0.335	0.694	0.212
Soy milk	0.92(0.84,1.01)	0.085	0.565	1.01(0.91,1.13)	0.785	0.909	0.198
Meat substitutes - soy	0.97(0.89,1.07)	0.542	0.892	1.02(0.91,1.13)	0.787	0.909	0.488
Spirits	0.98(0.92,1.05)	0.616	0.892	0.97(0.87,1.07)	0.500	0.836	0.870
Sushi	1.01(0.94,1.09)	0.749	0.986	1.02(0.92,1.14)	0.689	0.889	0.882
Other sweets	1.00(0.93,1.07)	0.930	0.986	0.94(0.83,1.06)	0.329	0.694	0.390
Tea	0.96(0.89,1.03)	0.211	0.749	1.08(0.97,1.19)	0.166	0.511	0.066
Tea, decaffeinated	0.96(0.89,1.04)	0.297	0.875	0.92(0.81,1.05)	0.204	0.544	0.582
Tomatoes	1.03(0.96,1.10)	0.461	0.879	0.97(0.87,1.09)	0.649	0.885	0.372
Vegetable dips	0.97(0.90,1.05)	0.512	0.892	0.95(0.84,1.08)	0.416	0.782	0.782
Meat substitutes - vegetarian	1.01(0.94,1.09)	0.756	0.986	0.98(0.87,1.11)	0.797	0.909	0.678
Other vegetables	0.97(0.90,1.04)	0.410	0.878	0.94(0.83,1.05)	0.259	0.611	0.656
Vegetable side dishes	1.01(0.94,1.08)	0.837	0.986	0.80(0.68,0.94)	0.007	0.101	0.010
Water (still and sparkling)	1.07(1.00,1.15)	0.050	0.530	1.02(0.92,1.14)	0.699	0.889	0.464
White fish and tinned tuna	0.97(0.90,1.04)	0.338	0.875	0.98(0.88,1.09)	0.662	0.889	0.876
Whole milk	0.97(0.90,1.04)	0.373	0.878	1.06(0.98,1.15)	0.142	0.447	0.107
Fortified wine	0.99(0.93,1.06)	0.852	0.986	0.97(0.87,1.08)	0.584	0.846	0.752
Red wine	1.06(1.00,1.13)	0.063	0.530	1.08(0.98,1.18)	0.110	0.373	0.742
White wine	1.05(0.98,1.11)	0.170	0.677	1.00(0.90,1.11)	0.968	0.993	0.433
Cream	1.00(0.94,1.07)	0.925	0.986	1.02(0.93,1.13)	0.642	0.884	0.740
Trans fatty acids	1.00(0.92,1.09)	0.935	0.986	1.03(0.92,1.17)	0.584	0.846	0.694

CI, confidence interval; FDR, false discovery rate; HR, hazard ratio

All dietary factors entered the models as standardized continuous variables and reflect associations per 1-SD increase in daily consumption. The model was adjusted for age at recruitment (continuous), sex(male/female), Townsend deprivation index (TDI, continuous), education (College or University degree/other), ethnicity(white/others), family history of CRC (yes/no/unknown)), regular aspirin use (yes/no/unknown), bowel screening(yes/no/unknown), diabetes(yes/no/unknown), body mass index (BMI, kg/m2, continuous), physical activity (minutes per week, continuous), smoking (pack-years, continuous), and total energy intake (KJ/day, continuous).

Table S4 The Association of the 139 Food and Nutrient Intakes with Colorectal Cancer Risk by gender stratification.

Foods and Nutrients	Female			Male			P value for Heterogeneity
	HR (95%CI)	P	FDRP	HR (95%CI)	P	FDRP	
**Nutrients**							
Carbohydrate	0.94(0.82,1.08)	0.351	0.831	0.83(0.73,0.93)	0.002	0.033	0.183
Dietary fibre	0.93(0.84,1.02)	0.125	0.831	0.83(0.76,0.91)	0.000	0.005	0.092
Calcium	0.92(0.84,1.02)	0.117	0.831	0.86(0.78,0.94)	0.001	0.026	0.326
Magnesium	0.88(0.78,1.00)	0.044	0.618	0.84(0.75,0.93)	0.002	0.032	0.579
Phosphorus	0.88(0.78,1.00)	0.043	0.618	0.82(0.73,0.92)	0.001	0.026	0.415
Manganese	0.93(0.84,1.03)	0.149	0.831	0.84(0.77,0.91)	0.000	0.005	0.130
Alcohol	1.02(0.94,1.11)	0.597	0.919	1.12(1.05,1.19)	0.001	0.026	0.415
Protein	0.97(0.87,1.09)	0.613	0.919	1.00(0.90,1.11)	0.954	0.968	0.698
Vegetable protein	0.98(0.89,1.09)	0.764	0.974	0.88(0.80,0.97)	0.012	0.136	0.131
Animal protein	0.98(0.90,1.08)	0.745	0.974	1.05(0.96,1.14)	0.285	0.630	0.280
Fat	1.07(0.92,1.23)	0.372	0.835	0.98(0.86,1.11)	0.722	0.904	0.373
Vegetable fat	1.02(0.92,1.13)	0.736	0.974	0.95(0.87,1.04)	0.283	0.630	0.306
Animal fat	1.03(0.93,1.15)	0.525	0.904	1.02(0.93,1.13)	0.608	0.858	0.894
Saturated fatty acids	1.03(0.92,1.17)	0.573	0.919	1.00(0.90,1.12)	0.943	0.968	0.721
n-3 fatty acids	1.05(0.96,1.14)	0.307	0.831	0.93(0.86,1.02)	0.119	0.488	0.050
n-6 fatty acids	1.02(0.92,1.13)	0.718	0.974	0.94(0.85,1.03)	0.202	0.588	0.255
Iron	0.98(0.88,1.10)	0.767	0.974	0.93(0.83,1.03)	0.175	0.588	0.509
Vitamin B6	0.95(0.86,1.04)	0.261	0.831	1.02(0.93,1.11)	0.719	0.904	0.283
Vitamin B12	1.05(0.97,1.13)	0.233	0.831	0.97(0.90,1.05)	0.429	0.726	0.152
Folate	0.96(0.87,1.05)	0.336	0.831	0.94(0.86,1.02)	0.161	0.573	0.745
Vitamin C	1.01(0.93,1.10)	0.806	0.974	0.95(0.88,1.02)	0.164	0.573	0.283
Potassium	0.94(0.85,1.05)	0.265	0.831	0.91(0.82,1.00)	0.053	0.314	0.661
Retinol	1.08(1.02,1.14)	0.011	0.612	1.03(0.96,1.09)	0.420	0.726	0.271
Total carotene	1.00(0.92,1.08)	0.969	0.976	1.00(0.94,1.08)	0.903	0.963	1.000
Vitamin E	1.11(1.00,1.23)	0.057	0.662	0.92(0.83,1.01)	0.087	0.388	0.010
Vitamin D	1.04(0.96,1.12)	0.382	0.842	0.97(0.90,1.04)	0.395	0.713	0.196
Starch	1.01(0.90,1.13)	0.865	0.974	0.98(0.89,1.08)	0.645	0.862	0.692
Monounsaturated fatty acids	1.07(0.94,1.22)	0.320	0.831	1.01(0.90,1.14)	0.864	0.943	0.520
Zinc	1.01(0.91,1.12)	0.835	0.974	0.94(0.85,1.03)	0.199	0.588	0.320
Thiamin	0.94(0.86,1.04)	0.243	0.831	0.92(0.84,1.00)	0.059	0.314	0.744
Riboflavin	0.94(0.85,1.03)	0.189	0.831	0.89(0.81,0.97)	0.008	0.106	0.416
Cholesterol	0.97(0.89,1.05)	0.438	0.882	1.04(0.96,1.12)	0.339	0.644	0.227
Alpha-carotene	1.01(0.93,1.09)	0.856	0.974	1.01(0.95,1.08)	0.684	0.904	1.000
Beta-carotene	1.00(0.93,1.08)	0.960	0.974	1.00(0.94,1.08)	0.920	0.963	1.000
Beta cryptoxanthin	0.93(0.85,1.03)	0.155	0.831	0.99(0.92,1.07)	0.834	0.943	0.316
Biotin	0.96(0.87,1.06)	0.443	0.882	0.93(0.85,1.02)	0.113	0.476	0.643
Chloride	1.04(0.93,1.16)	0.496	0.904	1.09(0.98,1.21)	0.100	0.436	0.547
Copper	1.00(0.91,1.11)	0.954	0.974	0.95(0.87,1.05)	0.324	0.643	0.462

Iodine	0.91(0.83,0.99)	0.035	0.612	0.93(0.86,1.01)	0.074	0.344	0.721
Sodium	1.06(0.95,1.17)	0.290	0.831	1.10(1.00,1.21)	0.046	0.303	0.607
Niacin equivalent	1.01(0.91,1.12)	0.821	0.974	1.02(0.92,1.12)	0.767	0.904	0.893
Pantothenic acid	0.92(0.83,1.02)	0.109	0.831	0.91(0.83,0.99)	0.037	0.269	0.875
Selenium	0.97(0.89,1.06)	0.471	0.897	0.97(0.90,1.05)	0.434	0.726	1.000
Total nitrogen	0.97(0.86,1.09)	0.600	0.919	0.97(0.87,1.08)	0.575	0.858	1.000
Vitamin A retinol equivalents	1.08(1.01,1.15)	0.028	0.612	1.03(0.96,1.09)	0.429	0.726	0.306
**Foods**							
White bread	1.09(1.01,1.17)	0.027	0.612	1.12(1.05,1.19)	0.001	0.026	0.582
Processed meat	1.02(0.94,1.10)	0.662	0.930	1.10(1.03,1.17)	0.003	0.050	0.144
Animal fat spread lower fat	1.00(0.92,1.08)	0.946	0.974	0.99(0.93,1.06)	0.868	0.943	0.849
Animal fat spread normal	1.02(0.94,1.10)	0.627	0.919	0.99(0.92,1.06)	0.777	0.908	0.580
Added sugars and preserves	0.95(0.88,1.04)	0.273	0.831	0.98(0.91,1.05)	0.587	0.858	0.580
Allium vegetables	0.99(0.92,1.08)	0.897	0.974	0.96(0.90,1.03)	0.296	0.630	0.565
Beef	1.09(1.01,1.17)	0.019	0.612	0.99(0.92,1.06)	0.742	0.904	0.065
Beer and cider	0.93(0.84,1.04)	0.204	0.831	1.08(1.01,1.15)	0.024	0.216	0.019
Biscuits	0.94(0.87,1.03)	0.179	0.831	1.04(0.97,1.11)	0.251	0.630	0.067
Breaded/battered chicken	1.04(0.96,1.11)	0.335	0.831	1.01(0.95,1.09)	0.702	0.904	0.566
Breaded/battered fish	0.92(0.83,1.01)	0.070	0.746	0.96(0.89,1.03)	0.247	0.630	0.495
Mixed bread (50/50), brown and seeded	1.01(0.93,1.09)	0.867	0.974	0.98(0.92,1.05)	0.611	0.858	0.567
Other bread	1.01(0.94,1.10)	0.733	0.974	0.96(0.88,1.03)	0.261	0.630	0.371
Whole meal bread	0.97(0.90,1.05)	0.454	0.882	0.92(0.85,0.99)	0.025	0.216	0.339
Biscuit cereal	1.00(0.92,1.08)	0.910	0.974	0.98(0.91,1.05)	0.519	0.810	0.713
Bran cereal	0.97(0.89,1.05)	0.438	0.882	0.91(0.84,0.98)	0.013	0.136	0.268
Oat cereal (non sugar)	0.96(0.89,1.04)	0.353	0.831	0.93(0.87,1.00)	0.057	0.314	0.551
Oat cereal (sugar)	0.99(0.91,1.07)	0.777	0.974	0.96(0.89,1.04)	0.294	0.630	0.591
Other cereal (sugar)	1.03(0.96,1.11)	0.392	0.852	1.03(0.97,1.10)	0.328	0.643	1.000
Chocolate confectionery	1.05(0.97,1.14)	0.217	0.831	1.00(0.92,1.07)	0.917	0.963	0.387
Coffee, caffeinated	0.99(0.92,1.08)	0.895	0.974	1.02(0.95,1.09)	0.632	0.861	0.580
Coffee, decaffeinated	0.99(0.92,1.07)	0.760	0.974	0.91(0.84,0.98)	0.015	0.153	0.126
Savoury crackers	0.96(0.88,1.04)	0.285	0.831	0.99(0.92,1.06)	0.751	0.904	0.582
Milk-dairy desserts	1.01(0.93,1.09)	0.870	0.974	0.97(0.90,1.04)	0.327	0.643	0.461
Other desserts and cakes and pastries	0.99(0.92,1.08)	0.878	0.974	0.98(0.91,1.05)	0.499	0.798	0.853
Soy desserts and yogurt	1.01(0.95,1.09)	0.685	0.952	0.95(0.87,1.04)	0.272	0.630	0.287
Milk-based and powdered drinks	1.00(0.92,1.08)	0.926	0.974	1.00(0.94,1.07)	0.974	0.974	1.000
Egg and egg dishes	0.94(0.87,1.03)	0.171	0.831	1.00(0.93,1.07)	0.964	0.971	0.269
Apples and pears	0.90(0.83,0.98)	0.015	0.612	0.95(0.88,1.02)	0.125	0.495	0.340
Berries	1.00(0.92,1.07)	0.902	0.974	0.99(0.92,1.06)	0.749	0.904	0.849
Citrus	1.02(0.95,1.10)	0.537	0.904	0.98(0.91,1.04)	0.471	0.772	0.429
Dried fruit	0.99(0.91,1.06)	0.708	0.974	0.93(0.86,1.00)	0.057	0.314	0.253
Other fruit	0.96(0.89,1.04)	0.324	0.831	0.92(0.86,0.99)	0.027	0.222	0.427

Stewed fruit	1.00(0.93,1.07)	0.951	0.974	0.97(0.90,1.03)	0.303	0.630	0.540
Fruit juice	1.06(0.99,1.15)	0.111	0.831	0.96(0.90,1.04)	0.328	0.643	0.062
Full fat yogurt	0.97(0.90,1.06)	0.516	0.904	1.04(0.97,1.11)	0.246	0.630	0.198
Grain dishes - added fat	0.96(0.89,1.05)	0.399	0.854	1.01(0.95,1.08)	0.717	0.904	0.342
Green leafy/cabbages	1.02(0.95,1.10)	0.607	0.919	0.98(0.92,1.05)	0.611	0.858	0.427
High fat cheese	1.05(0.97,1.14)	0.228	0.831	0.98(0.91,1.06)	0.628	0.861	0.224
Lamb	0.97(0.90,1.05)	0.457	0.882	1.06(1.00,1.13)	0.040	0.278	0.077
Legumes and pulses	0.94(0.86,1.02)	0.144	0.831	1.01(0.94,1.08)	0.821	0.943	0.201
Low fat yogurt	0.94(0.87,1.02)	0.152	0.831	0.95(0.88,1.02)	0.131	0.505	0.848
Medium and low fat cheese	0.92(0.84,1.00)	0.057	0.662	1.00(0.93,1.07)	0.897	0.963	0.144
Other meat, offal	0.94(0.86,1.03)	0.212	0.831	1.01(0.94,1.07)	0.866	0.943	0.205
Muesli	1.00(0.93,1.08)	0.957	0.974	0.97(0.90,1.04)	0.408	0.718	0.566
Nut-based spreads	1.08(1.01,1.15)	0.035	0.612	0.99(0.92,1.07)	0.866	0.943	0.087
Unsalted nuts and seeds	0.95(0.87,1.03)	0.210	0.831	0.95(0.88,1.03)	0.203	0.588	1.000
Salted nuts and seeds	0.98(0.90,1.07)	0.640	0.919	1.02(0.95,1.09)	0.640	0.862	0.478
Oily fish	1.04(0.97,1.12)	0.305	0.831	0.95(0.88,1.02)	0.165	0.573	0.085
Olive oil (drizzling/dunking)	1.03(0.96,1.11)	0.444	0.882	0.94(0.87,1.02)	0.153	0.573	0.096
Plant-based spread lower fat	1.04(0.97,1.12)	0.283	0.831	1.04(0.98,1.11)	0.184	0.588	1.000
Plant-based spread normal	1.04(0.97,1.12)	0.299	0.831	1.04(0.97,1.11)	0.259	0.630	1.000
White pasta and rice	0.99(0.91,1.08)	0.862	0.974	0.95(0.88,1.03)	0.231	0.630	0.487
Whole meal pasta, brown rice and other wholegrains	0.96(0.88,1.05)	0.370	0.835	0.96(0.88,1.04)	0.279	0.630	1.000
Peas and sweetcorn	0.99(0.92,1.08)	0.894	0.974	0.99(0.92,1.06)	0.751	0.904	1.000
Pizza	1.00(0.91,1.09)	0.926	0.974	1.02(0.95,1.10)	0.565	0.858	0.739
Pork	0.97(0.90,1.05)	0.479	0.899	1.00(0.93,1.07)	0.937	0.968	0.567
Potatoes and sweet potatoes (baked/boiled)	0.98(0.90,1.05)	0.536	0.904	1.01(0.95,1.08)	0.738	0.904	0.556
Fried/roast potatoes	1.01(0.93,1.09)	0.853	0.974	1.04(0.97,1.11)	0.274	0.630	0.582
Mashed potatoes	1.03(0.96,1.11)	0.413	0.869	1.02(0.96,1.09)	0.472	0.772	0.843
Poultry	1.00(0.92,1.08)	0.987	0.987	1.07(1.00,1.14)	0.056	0.314	0.200
Raw salad	0.99(0.91,1.07)	0.786	0.974	1.03(0.96,1.10)	0.406	0.718	0.463
Rice/oat milk	0.94(0.83,1.06)	0.317	0.831	0.78(0.50,1.21)	0.261	0.630	0.425
Root vegetables	0.96(0.89,1.04)	0.320	0.831	1.00(0.94,1.07)	0.921	0.963	0.430
Low/non sugar sugar-sweetened beverages	0.87(0.78,0.97)	0.013	0.612	1.06(1.00,1.13)	0.068	0.338	0.002
Sugar-sweetened beverages and other sugary drinks	1.02(0.94,1.11)	0.569	0.919	0.98(0.91,1.06)	0.601	0.858	0.487
Samosa, pakora	0.97(0.88,1.07)	0.521	0.904	0.99(0.92,1.07)	0.761	0.904	0.746
Sauces and condiments (high fat)	1.04(0.96,1.12)	0.330	0.831	1.06(0.99,1.13)	0.071	0.338	0.713
Sauces and condiments (low fat)	1.02(0.95,1.11)	0.567	0.919	0.96(0.89,1.03)	0.283	0.630	0.266
Semi skimmed milk	1.00(0.92,1.08)	0.931	0.974	0.96(0.89,1.02)	0.203	0.588	0.447
Shellfish	1.02(0.95,1.10)	0.539	0.904	1.03(0.97,1.09)	0.389	0.711	0.838
Skimmed milk and cholesterol-lowering milk	0.95(0.87,1.03)	0.176	0.831	0.96(0.89,1.03)	0.225	0.630	0.854
Savoury snacks	1.05(0.97,1.14)	0.245	0.831	1.03(0.96,1.11)	0.352	0.652	0.729
Soups	1.05(0.98,1.13)	0.188	0.831	1.03(0.97,1.10)	0.336	0.644	0.692

Soy milk	1.02(0.94,1.10)	0.648	0.919	0.91(0.83,1.01)	0.069	0.338	0.075
Meat substitutes - soy	0.99(0.91,1.09)	0.846	0.974	0.92(0.82,1.04)	0.186	0.588	0.335
Spirits	0.97(0.90,1.06)	0.531	0.904	0.98(0.92,1.04)	0.500	0.798	0.844
Sushi	1.05(0.98,1.11)	0.161	0.831	1.01(0.94,1.09)	0.766	0.904	0.431
Other sweets	0.97(0.89,1.06)	0.540	0.904	0.99(0.93,1.06)	0.829	0.943	0.714
Tea	0.96(0.88,1.04)	0.268	0.831	1.00(0.93,1.07)	0.954	0.968	0.463
Tea, decaffeinated	0.96(0.88,1.04)	0.310	0.831	0.95(0.88,1.02)	0.181	0.588	0.854
Tomatoes	1.02(0.94,1.10)	0.636	0.919	0.98(0.92,1.05)	0.594	0.858	0.445
Vegetable dips	0.98(0.90,1.06)	0.616	0.919	0.98(0.91,1.06)	0.621	0.861	1.000
Meat substitutes - vegetarian	1.05(0.98,1.12)	0.202	0.831	0.98(0.90,1.06)	0.589	0.858	0.200
Other vegetables	0.96(0.88,1.04)	0.291	0.831	0.97(0.90,1.04)	0.343	0.644	0.854
Vegetable side dishes	1.00(0.92,1.08)	0.943	0.974	0.91(0.84,0.99)	0.029	0.222	0.107
Water (still and sparkling)	1.04(0.96,1.13)	0.295	0.831	1.02(0.95,1.10)	0.505	0.798	0.729
White fish and tinned tuna	0.96(0.89,1.04)	0.365	0.835	0.98(0.91,1.05)	0.529	0.818	0.702
Whole milk	1.01(0.94,1.09)	0.808	0.974	1.01(0.94,1.08)	0.841	0.943	1.000
Fortified wine	1.02(0.95,1.09)	0.588	0.919	0.96(0.89,1.04)	0.303	0.630	0.253
Red wine	1.04(0.96,1.12)	0.346	0.831	1.09(1.02,1.15)	0.007	0.097	0.346
White wine	1.02(0.94,1.10)	0.641	0.919	1.04(0.97,1.10)	0.279	0.630	0.705
Cream	1.01(0.94,1.09)	0.742	0.974	0.99(0.93,1.06)	0.836	0.943	0.692
Trans fatty acids	1.02(0.93,1.12)	0.618	0.919	0.98(0.91,1.07)	0.709	0.904	0.525

CI, confidence interval; FDR, false discovery rate; HR, hazard ratio

All dietary factors entered the models as standardized continuous variables and reflect associations per 1-SD increase in daily consumption. The model was adjusted for age at recruitment (continuous), sex(male/female), Townsend deprivation index (TDI, continuous), education (College or University degree/other), ethnicity(white/others), family history of CRC (yes/no/unknown)), regular aspirin use (yes/no/unknown), bowel screening(yes/no/unknown), diabetes(yes/no/unknown), body mass index (BMI, kg/m2, continuous), physical activity (minutes per week, continuous), smoking (pack-years, continuous), and total energy intake (KJ/day, continuous).

Table S5 Risk of incident colorectal cancer according to genetic risk.

Genetic risk	Total participants	Cases (%)	Person-years	IR [a]	HR (95% CI) [b]	P value
Low	38431	274(19)	491787.7	0.56	Ref.	
Intermediate	38604	448(31)	493192.8	0.91	1.61(1.39,1.87)	4.77E-10
High	38863	706(50)	494945.8	1.43	2.55(2.21,2.93)	< 2e-16
per SD increase	115898	1428	1479926		1.54(1.46,1.62)	< 2e-16

IR incidence rate; HR hazard ratio; CI confidence interval

[a] Incidence rates are provided per 1000 person-years.

[b] The model was adjusted for age at recruitment (continuous), sex(male/female), Townsend deprivation index (TDI, continuous), education (College or University degree/other), ethnicity(white/others), family history of CRC (yes/no/unknown)), regular aspirin use (yes/no/unknown), bowel screening(yes/no/unknown), diabetes(yes/no/unknown), body mass index (BMI, kg/m2, continuous), physical activity (minutes per week, continuous), smoking (pack-years, continuous), and total energy intake (KJ/day, continuous).
